# Genetic-risk-informed integration of bulk and single-cell transcriptomes prioritizes *ANPEP* as a monocyte-associated candidate in aortic dissection

**DOI:** 10.3389/fcvm.2026.1965670

**Published:** 2026-10-07

**Authors:** Hongjiu Zhang, Manhong Yi, Xiong Zhang, Jiang Han, Keyi Fan, Runze Chang, Yongbin Shi

**Affiliations:** 1 Department of Vascular Surgery, The Second Hospital of Shanxi Medical University, Taiyuan, China; 2 Department of Nephrology, The Second Hospital of Shanxi Medical University, Taiyuan, China; 3 Department of Cardiology, Peking Union Medical College Hospital, Chinese Academy of Medical Sciences and Peking Union Medical College, Beijing, China

**Keywords:** ANPEP, aortic dissection, genetic susceptibility, monocytes, single-cell RNA sequencing, vascular inflammation

## Abstract

**Background:**

Aortic dissection is characterized by vascular wall remodeling and inflammatory activation, but the cell-associated molecular programs linking transcriptional alterations to genetic susceptibility remain incompletely defined. This study aimed to identify reproducible monocyte-associated molecular candidates in aortic dissection.

**Methods:**

We integrated bulk transcriptomic discovery (GSE153434) with an exploratory secondary-cohort assessment (GSE52093), single-cell RNA sequencing (GSE189795), FinnGen genome-wide association study summary statistics, scPagwas, and cell-type-specific hdWGCNA. *ANPEP* and *CCL20* transcriptional responses were subsequently assessed by droplet digital PCR in five independent paired experiments using angiotensin II-stimulated, PMA-differentiated THP-1 macrophage-like cells. Immune-context, cellular-state, compound-screening, and spatial analyses were performed as exploratory hypothesis-generating analyses.

**Results:**

Genetic risk-informed analysis highlighted myeloid populations, particularly monocytes and neutrophils. Integration of bulk differential expression, scPagwas correlations, and a disease-associated monocyte hdWGCNA module prioritized *ANPEP*, *CCL20*, and *CD82*. *ANPEP* showed the most consistent support across the analyzed evidence layers. In the secondary cohort GSE52093, ANPEP and CD82 expression levels were higher in aortic dissection samples, whereas CCL20 showed weaker cross-cohort consistency; these findings were interpreted as exploratory secondary-cohort evidence rather than clinical validation. Angiotensin II stimulation increased *ANPEP* and *CCL20* transcript abundance in THP-1 macrophage-like cells. Exploratory analyses further associated the prioritized genes with heterogeneous myeloid inflammatory states.

**Conclusion:**

Integrative genetic and transcriptomic analyses prioritized *ANPEP* as a comparatively consistent monocyte-associated candidate in aortic dissection. These results require confirmation in primary human cells, independent patient samples, and functional perturbation models.

## 1 Introduction

Aortic dissection is an acute, potentially fatal disease caused by the separation of the aortic wall layers (1–3). Despite advances in imaging, surgical management, and risk stratification, early recognition remains difficult (4). Mechanistic interpretation is also challenging because clinical presentation is heterogeneous, whereas disease progression may be abrupt (5). Current guidelines emphasize rapid diagnosis, anatomical classification, and timely intervention (6). More informative biomarkers are still needed to refine risk assessment and individualized management (7).

The pathological basis of aortic dissection involves structural weakening of the aortic media, loss of smooth muscle cell homeostasis, extracellular matrix degradation, and inflammatory activation. These events occur within a complex tissue microenvironment where vascular cells, immune cells, and matrix components interact dynamically (8–10). Clinical and experimental studies have linked aortic wall degeneration to vascular inflammation, immune cell infiltration, and remodeling of elastin- or collagen-rich matrix (11, 12). Transcriptional alterations in resident vascular cells or infiltrating immune cells may therefore contribute to disease initiation and progression.

Bulk transcriptomic studies have identified candidate genes and pathways for aortic dissection. However, averaging signals across mixed cell populations limits the resolution of cell-type-specific disease programs. Single-cell ribonucleic acid sequencing (scRNA-seq) assigns transcriptional changes to individual cellular compartments, including smooth muscle cells, endothelial cells, fibroblast-like cells, and immune-cell subsets (13). Such findings may still be affected by sample composition, annotation strategy, or dataset-specific technical factors. Integration with bulk expression, genetic association information, and a secondary-cohort assessment may help prioritize more robust disease-associated genes.

Monocytes and macrophage-lineage cells are of particular interest in aortic wall injury because they may connect systemic inflammation with local vascular remodeling (14). These cells participate in cytokine signaling, matrix degradation, and crosstalk with smooth muscle cells, processes relevant to aortic wall instability. Smooth muscle cells, meanwhile, remain central to the mechanical integrity of the aortic media (15, 16). Joint evaluation of immune-cell signals with smooth muscle cell modules may therefore provide a more balanced view than analysis of a single data layer or cell population.

In addition to gene prioritization, exploratory downstream analyses can provide hypothesis-generating links between disease-associated transcriptional states and candidate molecular perturbations (17, 18). Such analyses should be interpreted cautiously because predicted compound–gene or compound–signature relationships represent compound-screening hypotheses and do not establish biological activity or clinical benefit.

Given the contribution of monocyte/macrophage activation to aortic wall injury, we hypothesized that integration of bulk, single-cell, and genetic evidence would identify reproducible monocyte-associated molecular candidates in aortic dissection. We therefore integrated bulk transcriptomic, GWAS-informed single-cell, and cell-type-specific co-expression evidence, followed by exploratory assessment in a secondary cohort and preliminary THP-1 ddPCR assessment. The primary objective was molecular candidate prioritization rather than establishment of a clinical diagnostic model or therapeutic mechanism. Immune-context, cellular-state, spatial, and compound-screening analyses were included as exploratory hypothesis-generating components (Figure 1).

**Figure 1:**
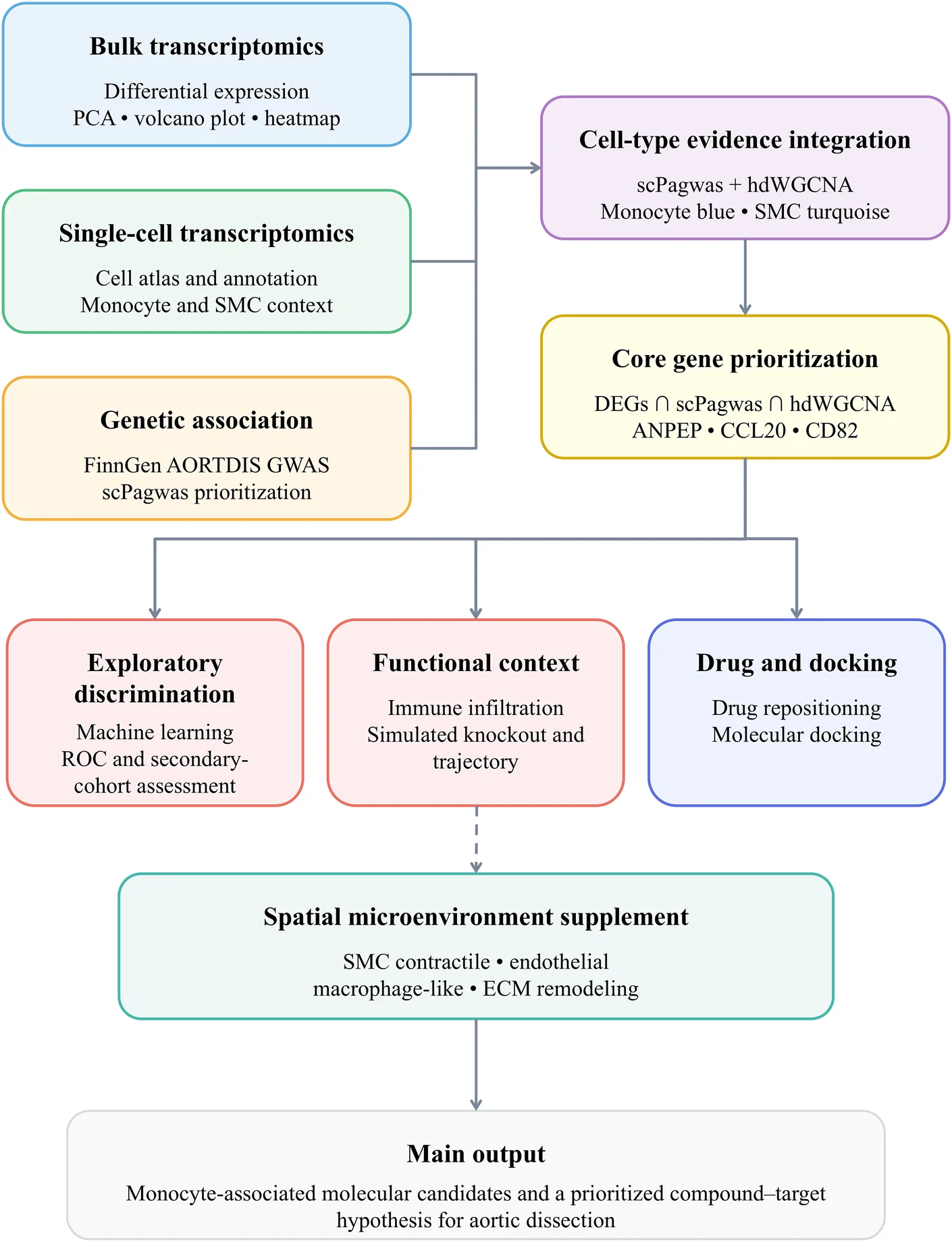
Integrative multi-omics study workflow. Overview of the analytical framework used in this study. Bulk transcriptomic analysis, single-cell transcriptomic analysis, and genetic association information were integrated to identify disease-associated cell-type contexts and prioritize core candidate genes. The prioritized candidates were further assessed through exploratory secondary-cohort assessment and preliminary THP-1 ddPCR experiments, followed by exploratory machine-learning discrimination, immune-context, cellular-state, compound-screening, molecular-docking, and spatial microenvironment analyses.

## 2 Materials and methods

### 2.1 Study design and data sources

The primary objective of this study was to identify reproducible monocyte-associated inflammatory signatures in aortic dissection by integrating bulk, single-cell, genetic, and cell-type-specific co-expression evidence.

This study integrated publicly available bulk transcriptomic, single-cell RNA sequencing (scRNA-seq), genome-wide association study (GWAS), and spatial transcriptomic datasets. GSE153434 was used as the discovery cohort (19), whereas GSE52093 was used for exploratory secondary-cohort assessment (20). GSE189795 was used for scRNA-seq analysis (21), and aortic dissection GWAS summary statistics were obtained from FinnGen (22). GSE241870 was included to provide supplementary spatial information about the vascular microenvironment (23). All datasets were publicly available and de-identified. The datasets used in this study are summarized in Supplementary Table S1.

### 2.2 Bulk transcriptomic and functional enrichment analyses

Bulk transcriptomic data were preprocessed and annotated at the gene level. Differential expression analysis was performed using the limma package. Genes with |log2 fold change| > 0.5 and an adjusted *p*-value < 0.05 were considered differentially expressed genes. Gene Ontology and Kyoto Encyclopedia of Genes and Genomes enrichment analyses were performed using clusterProfiler.

### 2.3 Single-cell transcriptomic analysis

The scRNA-seq data were processed using Seurat. Cells were retained if they had at least 200 detected genes; no upper cutoffs for detected genes or total transcript counts were applied. Genes detected in fewer than 10 cells were excluded during Seurat object creation. Canonical mitochondrial genes were not detected in the processed matrix, so mitochondrial transcript proportion was not used for filtering. Ambient RNA contamination was assessed with decontX using sample-specific adaptive thresholds defined as Q3 + 1.5  ×  IQR and constrained to 0.10–0.30. Potential doublets were evaluated separately for each sample; samples with fewer than 100 cells were processed with scDblFinder, whereas larger samples used DoubletFinder with PCs 1–10, pN = 0.25, data-driven pK selection by maximal BCmetric, and homotypic-adjusted expected doublet numbers. Counts were then log-normalized with a scale factor of 10,000, and 1,500 highly variable genes were selected using the VST method. PCA was calculated using 20 components, and Harmony was performed with sample_id as the grouping variable; PCs 1–13 from the Harmony reduction were used for graph-based clustering and UMAP. The final clustering resolution was 0.3, with a diagnostic sweep from 0.1 to 1.0 in increments of 0.1. Cell types were annotated at the cluster level using SingleR with HumanPrimaryCellAtlasData and the reference dataset's label.main labels, and cluster labels were mapped to individual cells. For the ANPEP-focused audit, log-normalized ANPEP values were summarized across the existing broad cell-type annotations and by donor; these analyses were descriptive and did not treat individual cells as independent biological replicates.

### 2.4 Genetic-risk integration and gene prioritization

GWAS summary statistics were integrated with the scRNA-seq data using scPagwas. The analysis used the RNA assay, the hg37 genome build, a MAF filter of 0.01, marg = 10,000, a minimum of 10 cells per cluster, pathway sizes of 10–120 genes, 50 cell-type iterations, 25 single-cell iterations, 400 top genes, eight cores, and seed 123. The analysis was run without split mode and with single-cell and cell-type scoring enabled; outlier removal and Seurat-object return were enabled. Reference resources were loaded from the scPagwas installation, including the KEGG gene–pathway mapping, LD structure, and hg37 block annotation. Cell-type-specific co-expression networks were subsequently constructed using hdWGCNA for monocytes and smooth muscle cells. For hdWGCNA, genes were selected using the top 5% within-cell-type expression fraction, and metacells were constructed with k = 25 and max_shared = 10. Signed networks were built after selecting the soft-thresholding power by the SFT.R² criterion (≥0.8 or the maximum observed value); the saved monocyte and smooth muscle cell analyses used powers 6 and 7, respectively, with deepSplit = 4, minModuleSize = 50, and mergeCutHeight = 0.2. Candidate genes were prioritized by integrating bulk differentially expressed genes, scPagwas-associated genes, and genes from disease-relevant hdWGCNA modules.

### 2.5 Exploratory discrimination analysis and functional interpretation

Historically, the supplied pipeline evaluated 175 planned combinations involving Lasso, Ridge, elastic-net models with alpha values 0.2, 0.4, 0.5, 0.6, and 0.8, forward stepwise generalized linear models, glmBoost, plsRglm, support vector machines, linear discriminant analysis, quadratic discriminant analysis, k-nearest neighbors, naive Bayes, gradient boosting, XGBoost, AdaBoost, and random forest, including selector–classifier combinations. The saved prediction matrix contained 138 model columns rather than 175 successful fits; the exact historical hyperparameter grids could not be fully reconstructed from the supplied records. Complete model-by-cohort evaluations used all 20 samples in GSE153434 (10 controls and 10 aortic dissection samples) and all 12 samples in GSE52093 (5 controls and 7 aortic dissection samples); 12 plsRglm-related model-by-cohort cells were incomplete and yielded no predictions. In the historical workflow, feature selection was performed on the full discovery dataset before model fitting. Internal cv.glmnet and caret tuning steps were not nested within an outer resampling loop, and nested cross-validation was not performed. These historical outputs were therefore not used as confirmatory diagnostic evidence. For the revision, ANPEP, CCL20, and CD82 were fixed before resampling from the upstream candidate set, and no within-fold feature selection was performed. Standardization was fitted within each training fold. A prespecified L2-penalized logistic regression model was evaluated using nested leave-one-out cross-validation in GSE153434, with inner three-fold stratified cross-validation over C = 0.01, 0.1, 1, and 10. The model was then fitted once on all GSE153434 samples with prespecified C = 1.0 and assessed once in GSE52093; GSE52093 was not used for feature selection, hyperparameter tuning, or model choice. These analyses were interpreted as exploratory dataset-level and secondary-cohort discrimination, not clinical diagnostic validation.

### 2.6 Exploratory compound screening and molecular docking

Disease-associated transcriptional signatures were analyzed using DrugReflector to identify hypothesis-level compounds predicted to modulate the disease-associated expression profile. Compounds were selected for molecular docking according to their screening results, biological relevance, and structural availability. Molecular docking of the selected compounds with the prioritized molecular candidates was performed using AutoDock. Predicted binding energies and representative binding poses were used to compare predicted compound–target interactions.

### 2.7 Spatial transcriptomic analysis

GSE241870 was analyzed to characterize spatial variation in smooth muscle cell, endothelial, macrophage-like, and extracellular matrix-related signals. Module scores were calculated using available marker genes and visualized according to spatial coordinates. Because the targeted panel did not contain all core genes, this dataset was used only as supplementary evidence for vascular microenvironment heterogeneity.

### 2.8 THP-1 cell culture and preliminary ddPCR assessment

THP-1 cells (Procell, Wuhan, China; catalog no. CL-0233; RRID: CVCL_0006) were cultured in RPMI-1640 medium supplemented with 10% fetal bovine serum (Cellmax, Beijing, China, SA301.01) and 1% penicillin-streptomycin (Cellmax, Beijing, China, CPS101.02). Cells were differentiated with phorbol 12-myristate 13-acetate (PMA; Merck Millipore, Darmstadt, Germany, 524400) at 100 nM for 24 h, rested for 24 h, and then treated with angiotensin II (Ang II; MCE, Shanghai, China, HY-13948) at 1 μM for 24 h. Untreated PMA-differentiated cells served as controls.

Droplet digital PCR (ddPCR) was performed using the QX200 system (Bio-Rad, Hercules, CA, USA). Total RNA was extracted using the ME5 Universal Plus RNA Kit (Mei5bio, Beijing, China, MF036-01) and reverse-transcribed with PrimeScript RT Master Mix (Takara, Kusatsu, Japan, RR036A). Each 22 µL ddPCR reaction contained 11 µL QX200 ddPCR EvaGreen Supermix (Bio-Rad, 1864034), 100 nM primers, and 50–100 ng cDNA. Droplets were generated automatically, and PCR amplification was performed under the following conditions: 95 °C for 5 min; 40 cycles of 95 °C for 30 s and 60 °C for 40 s; 4 °C for 5 min; and 90 °C for 5 min. Absolute copy numbers were calculated using QuantaSoft software based on the Poisson distribution. *CCL20* and *ANPEP* expression levels were normalized to *GAPDH*. Primer sequences are listed in Supplementary Table S2. Five independent paired biological experiments were performed.

### 2.9 Statistical analysis

Statistical analyses were conducted using R and Python. Multiple-testing correction was performed using the Benjamini-Hochberg method where appropriate. Pearson or Spearman correlation analysis was used according to the analysis design. Unless otherwise stated, two-sided *P* values < 0.05 were considered statistically significant. Detailed software versions and major analysis parameters are provided in Supplementary Table S3. For the preliminary ddPCR assessment, five independent biological experiments were performed. In each experiment, PMA-differentiated cells from the same culture preparation were divided into control and Ang II-treated conditions, generating five matched pairs. Normality of the paired differences was assessed using the Shapiro–Wilk test. Comparisons were performed using a two-sided paired t-test when the paired differences were approximately normally distributed and a two-sided Wilcoxon signed-rank test otherwise. Data are presented as mean ± SD from five independent biological experiments.

## 3 Results

### 3.1 Bulk transcriptomic analysis identified aortic dissection-associated differentially expressed genes

To define the global transcriptional alterations associated with aortic dissection, we first analyzed bulk transcriptomic profiles from the discovery cohort. Principal component analysis showed a clear separation between the aortic dissection and control samples, indicating distinct global transcriptomic profiles between the two groups (Figure 2A). Differential-expression analysis identified 1,981 differentially expressed genes, including 941 upregulated and 1,040 downregulated genes. The volcano plot illustrated the magnitude and statistical significance of these transcriptional alterations (Figure 2B). Hierarchical clustering of the representative differentially expressed genes further demonstrated distinct expression patterns between the aortic dissection and control samples (Figure 2C). Gene Ontology and Kyoto Encyclopedia of Genes and Genomes enrichment analyses implicated hypoxia response, extracellular matrix organization, cell-substrate adhesion, focal adhesion, integrin signaling, HIF-1 signaling, PI3K-Akt signaling, MAPK signaling, and fluid shear stress-related pathways (Figure 2D). Collectively, these results indicated a disease-associated transcriptional state characterized by vascular wall remodeling, cellular stress, and inflammatory activation.

**Figure 2:**
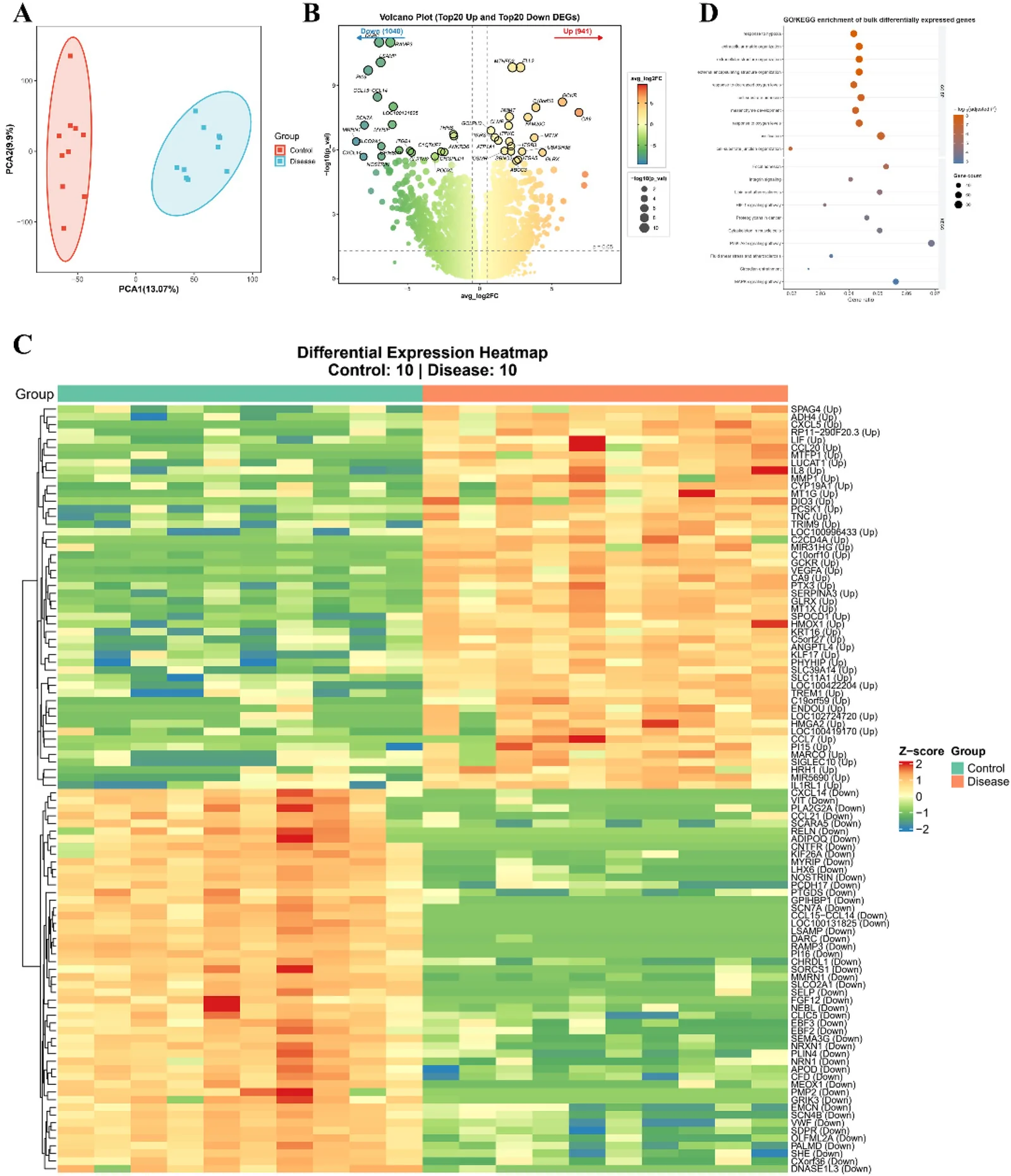
Bulk transcriptomic analysis and functional enrichment. **(A)** Principal component analysis of bulk transcriptomic samples from the discovery cohort showing global separation between aortic dissection and control samples. **(B)** Volcano plot showing differentially expressed genes between aortic dissection and control samples. **(C)** Heatmap of representative differentially expressed genes across samples. **(D)** Functional enrichment analysis of differentially expressed genes, highlighting biological processes and pathways related to vascular remodeling, extracellular matrix organization, cellular stress, and inflammatory activation. The discovery cohort included 10 control and 10 aortic dissection samples. Differentially expressed genes were defined using |log2 fold change| > 0.5 and a Benjamini–Hochberg-adjusted *p*-value < 0.05.

### 3.2 Single-cell analysis characterized the cellular context of aortic dissection

To resolve the cellular context underlying the bulk transcriptomic changes, we next analyzed single-cell RNA-seq data from normal and dissected ascending aortic tissues. Quality-control assessment showed the distributions of detected genes and transcript counts before filtering, together with a summary of cell and gene retention after filtering. DoubletFinder-based classification was used to identify potential doublets, and Harmony integration reduced sample-associated variation in the low-dimensional representation (Supplementary Figure S1). The scRNA-seq analysis resolved cell-type-level transcriptional features. Following quality control, normalization, dimensionality reduction, and clustering, major vascular and immune-cell populations were annotated, including endothelial cells, monocytes, myelocytes, neutrophils, natural killer cells, smooth muscle cells, and T cells (Figure 3A). Comparison of the control and aortic dissection groups showed that the major annotated cell populations were represented in both conditions, although their relative distributions differed between groups (Figure 3B). Highly variable feature selection and clustering-resolution assessment supported the dimensionality-reduction and clustering strategy. Marker-gene expression patterns were consistent with the assigned cell identities, while cell-type proportion analysis indicated condition-associated differences in the relative abundance of several cell populations (Supplementary Figure S2). In particular, immune-cell populations and smooth muscle cells showed condition-associated differences, suggesting concurrent inflammatory and vascular structural alterations in aortic dissection. Monocytes and smooth muscle cells were therefore selected as the principal cellular contexts for subsequent genetic risk-informed and co-expression analyses.

**Figure 3:**
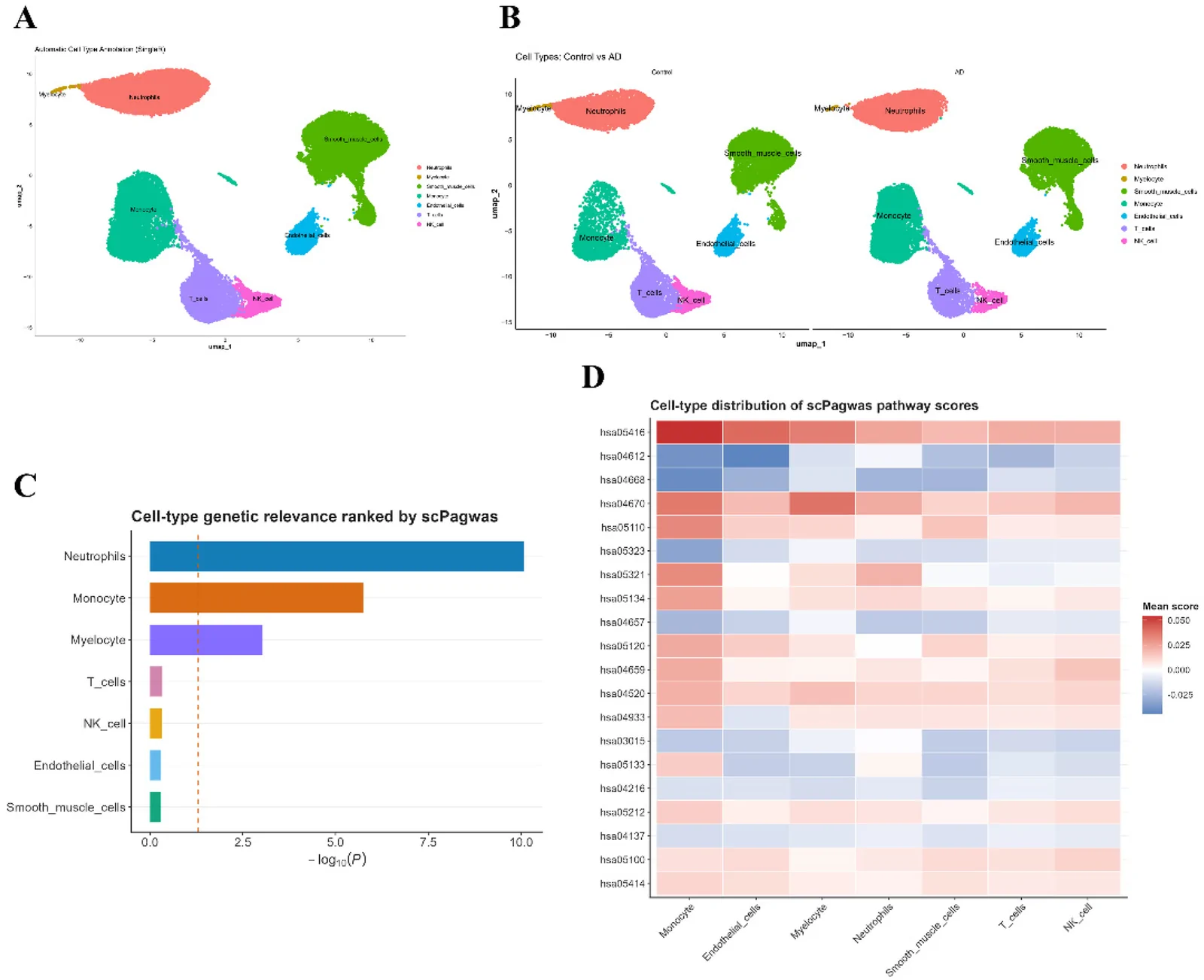
Single-cell landscape and genetic risk-informed analysis. **(A)** Uniform manifold approximation and projection (UMAP) visualization of annotated single-cell populations in ascending aortic tissue. **(B)** UMAP visualization stratified by disease group, showing the distribution of control and aortic dissection cells across annotated cell populations. **(C)** scPagwas-based cell-type association analysis integrating genetic association information with single-cell transcriptional profiles. **(D)** Cell-type-level pathway activity patterns across major vascular and immune-cell populations. The single-cell dataset comprised four control and five aortic dissection samples.

### 3.3 Genetic risk-informed analysis and hdWGCNA prioritized monocyte and smooth muscle cell modules

To connect disease-associated transcriptional programs with genetic susceptibility and cell-type-specific co-expression structure, we integrated GWAS-informed single-cell analysis with hdWGCNA. GWAS summary statistics were integrated with the single-cell profile using scPagwas to connect genetic susceptibility with cellular transcriptional states. The cell-type association analysis highlighted neutrophils and monocytes as the most prominent genetically relevant populations (Figure 3C). Cell-type-level pathway analysis further revealed heterogeneous pathway-activity patterns across monocytes, endothelial cells, myelocytes, neutrophils, smooth muscle cells, T cells, and natural killer cells (Figure 3D). Additional scPagwas analyses showed the distributions of gene-level pathway activation scores and trait-relevance scores across the single-cell landscape. Significant risk-associated cells were not uniformly distributed among cell types, and cell-type association statistics, bootstrap estimates, and gene-level PCC distributions provided complementary support for the genetically informed prioritization (Supplementary Figure S3).

The monocyte blue module contained 1,611 genes, whereas the smooth muscle cell turquoise module contained 2,252 genes. Cell-type-specific hdWGCNA identified multiple co-expression modules in monocytes and smooth muscle cells. The monocyte blue module and the smooth muscle cell turquoise module were selected for downstream integration based on their module characteristics and disease relevance (Figures 4A,B). Soft-threshold selection supported the network-construction parameters used for monocytes and smooth muscle cells. Module–cell-type association plots and hub-gene networks further characterized the selected co-expression modules and their principal network components (Supplementary Figure S4). In monocytes, integration of the blue-module genes, upregulated bulk DEGs, and genes with positive scPagwas PCC values identified 90 genes supported by all three evidence layers (Figure 4C). In smooth muscle cells, integration of the turquoise-module genes, downregulated bulk DEGs, and genes with negative scPagwas PCC values similarly identified 90 genes supported by the three corresponding evidence layers (Figure 4D). These directionally consistent intersections provided focused candidate sets for subsequent gene prioritization. Although both three-way intersections contained 90 genes, their gene memberships were distinct, with no overlap between the two lists; the identical cardinality was therefore coincidental and resulted from different module memberships, DEG-direction filters, and scPagwas PCC-sign criteria. The complete gene lists are provided in Supplementary Table S5.

**Figure 4:**
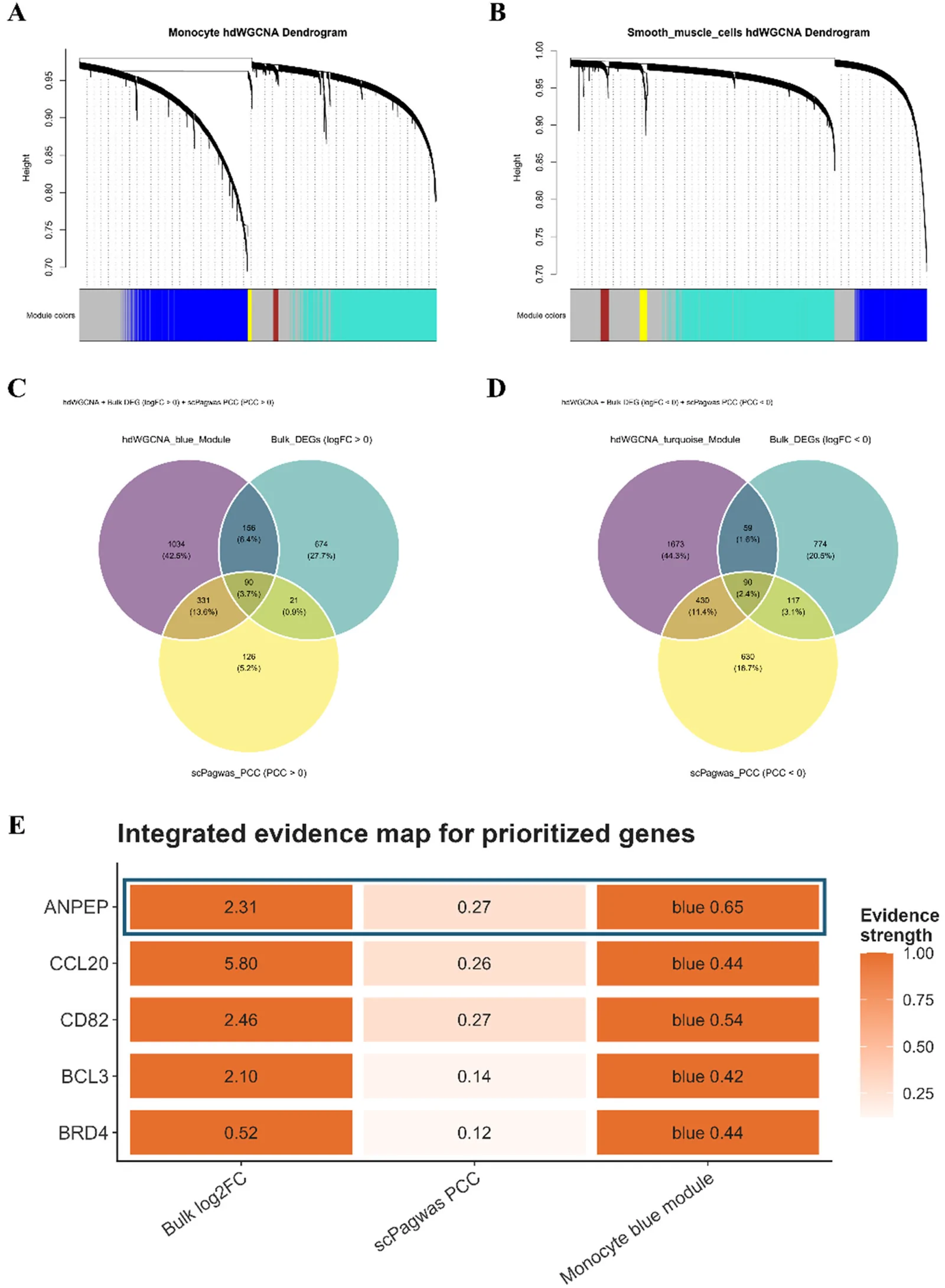
hdWGCNA and hdWGCNA and discovery-only gene prioritization. **(A,B)** hdWGCNA dendrograms and module assignments for monocytes and smooth muscle cells, respectively. **(C)** Intersection of monocyte blue-module genes, upregulated bulk differentially expressed genes (log2FC > 0), and genes with positive scPagwas gene-level PCC. **(D)** Intersection of smooth-muscle-cell turquoise-module genes, downregulated bulk differentially expressed genes (log2FC < 0), and genes with negative scPagwas gene-level PCC. Each three-way intersection contained 90 genes. Although the counts were identical, the gene memberships were distinct, with no overlap between the two lists. Complete directional intersection lists are provided in Supplementary Table S5. **(E)** Discovery-only evidence matrix summarizing bulk log2 fold change, scPagwas gene-level PCC, and monocyte blue-module membership for the candidate genes. ANPEP is highlighted for visual emphasis. GSE52093 secondary-cohort expression and ROC results were excluded from this matrix and are reported separately as exploratory secondary-cohort assessments. The matrix summarizes evidence layers and does not represent a formal learned rank score or clinical validation.

### 3.4 Cross-layer integration prioritized *ANPEP* as the leading monocyte-associated candidate

To identify robust candidate genes supported by multiple evidence layers, we intersected bulk differential expression, genetically informed single-cell signals, and cell-type-specific co-expression modules. Alanyl aminopeptidase, membrane (*ANPEP*; also known as aminopeptidase N or *CD13*), *CCL20*, and *CD82* were retained as the prioritized molecular candidates because the monocyte-centered integration supported them and showed consistent downstream relevance. *ANPEP* encodes a membrane-associated ectoenzyme expressed in several myeloid-cell populations. All three occurred among the positive-PCC genes with upregulated bulk expression in the monocyte blue module intersection. Their log2 fold changes (logFCs) were 2.31 for *ANPEP*, 5.80 for *CCL20*, and 2.46 for *CD82*. An integrated evidence matrix summarized bulk differential expression, scPagwas support, and monocyte blue-module membership for the prioritized genes (Figure 4E). The matrix was restricted to discovery evidence and did not include GSE52093 expression or ROC results. Within these discovery layers, *ANPEP* showed the most coherent profile and was therefore designated the leading candidate. *CCL20* and *CD82* were retained as supporting candidates because their evidence profiles differed in cross-cohort consistency and experimental support. *BCL3* and *BRD4* were evaluated only as auxiliary genes in supplementary exploratory analyses. Detailed evidence for these candidates is provided in Supplementary Table S4.

To further characterize the cellular context of ANPEP, we performed a descriptive audit of the preserved GSE189795 single-cell object. Across 35,760 cells from nine donors, ANPEP showed the highest mean log-normalized expression and fraction of expressing cells in the existing Monocyte annotation (mean 1.236; 52.6%), followed by Neutrophils (0.832; 27.0%) (Supplementary Figure S11). Donor-by-cell-type summaries were presented descriptively and showed donor heterogeneity. Thus, ANPEP-positive cells were enriched in the broad Monocyte compartment but were not monocyte-exclusive. Because the available annotations were broad and did not resolve classical or non-classical monocytes or macrophage subtypes, these data do not establish assignment to a specific monocyte/macrophage subtype. We also did not infer an inflammatory macrophage signature without a prespecified signature gene set.

### 3.5 Angiotensin II stimulation increased *ANPEP* and *CCL20* transcripts in THP-1 macrophage-like cells

To obtain preliminary author-generated experimental support for the prioritized inflammatory candidates, we quantified *ANPEP* and *CCL20* transcripts in five independent paired experiments using PMA-differentiated THP-1 macrophage-like cells. Angiotensin II stimulation significantly increased *CCL20* transcript abundance and produced a moderate but significant increase in *ANPEP*. These findings demonstrated transcriptional responsiveness in this *in vitro* inflammatory model but did not establish causal function or reproduce the findings in primary cells or patient tissue (Supplementary Figure S5).

### 3.6 Controlled exploratory discrimination and secondary-cohort assessment of a fixed three-gene model

The historical multialgorithm outputs were not used as confirmatory diagnostic evidence because feature selection occurred before resampling, some model units were incomplete, AUCs were inconsistent with saved predictions, and nested cross-validation was not performed. In the historical workflow, GSE52093 expression evidence entered the integrated evidence matrix before the present fixed-gene rerun and was subsequently reused for cohort-level discrimination analysis. Thus, GSE52093 was not a fully untouched validation cohort with respect to the complete historical pipeline. We therefore conducted a controlled exploratory rerun using a fixed three-gene set comprising ANPEP, CCL20, and CD82. For the revision, the discovery evidence map was separated from the secondary-cohort assessment, and the Figure 4E matrix was restricted to discovery evidence. The genes were fixed before resampling from the upstream candidate set; no within-fold feature selection was performed, and standardization was fitted within each training fold. In GSE153434 (*n* = 20; 10 controls and 10 aortic dissection samples), nested leave-one-out cross-validation with inner three-fold selection of C (0.01, 0.1, 1, and 10) yielded an AUC of 0.85 (bootstrap 95% interval, 0.64–1.00). These results are shown in Figure 5A and summarized in Supplementary Table S3.

**Figure 5:**
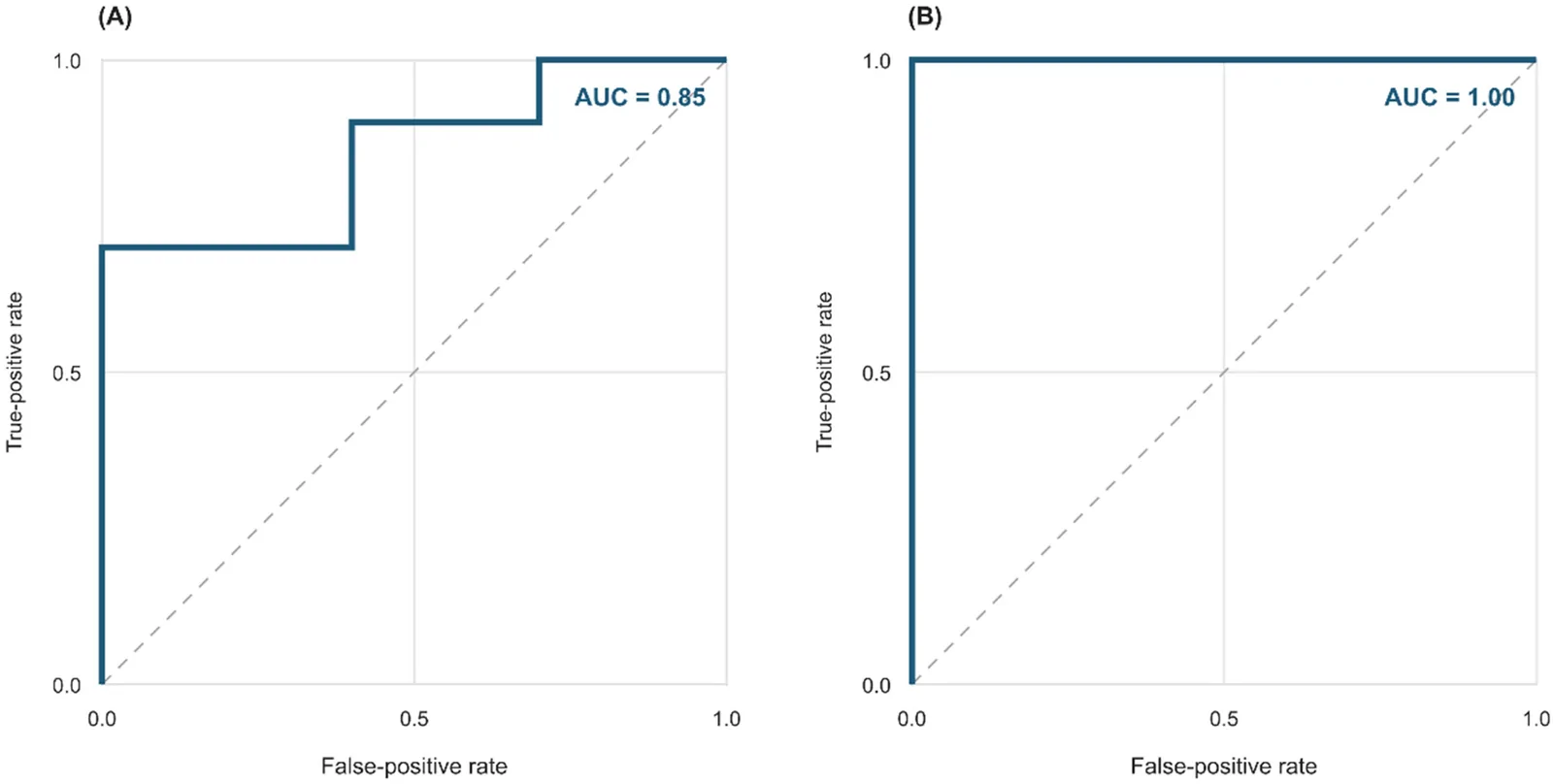
Controlled exploratory discrimination of a fixed three-gene model and secondary-cohort assessment. ANPEP, CCL20, and CD82 were fixed before resampling from the upstream candidate set. **(A)** Receiver operating characteristic curve from nested leave-one-out cross-validation in the GSE153434 discovery cohort, comprising 10 controls and 10 aortic dissection samples (*n* = 20). Standardization was fitted within each training fold, and the L2 logistic-regression parameter C was selected by inner three-fold stratified cross-validation from 0.01, 0.1, 1, and 10. The discovery-cohort AUC was 0.85 (bootstrap 95% interval, 0.64–1.00). **(B)** Receiver operating characteristic curve from one assessment of the model fitted on all GSE153434 samples with prespecified C = 1.0 and evaluated in the GSE52093 secondary cohort, comprising 5 controls and 7 aortic dissection samples (*n* = 12). The secondary-cohort AUC was 1.00 (bootstrap 95% interval, 1.00–1.00). GSE52093 had contributed to the historical integrated evidence matrix but was not used for feature selection, hyperparameter tuning, or model choice in the revised fixed-gene rerun. Both panels are exploratory and do not constitute clinical diagnostic validation; upstream candidate selection may still introduce optimism.

The model was then fitted once on all GSE153434 samples with prespecified C = 1.0 and assessed once in the GSE52093 secondary cohort (*n* = 12; 5 controls and 7 aortic dissection samples), yielding an AUC of 1.00 (bootstrap 95% interval, 1.00–1.00; Figure 5B). Because GSE52093 had contributed to the historical integrated evidence matrix, the present result is described as a secondary-cohort exploratory assessment rather than a fully independent validation. In the revised fixed-gene rerun, GSE52093 was not used for feature selection, hyperparameter tuning, or model choice. Given the small sample size and upstream candidate selection, this result was interpreted only as exploratory secondary-cohort discrimination and not as clinical diagnostic validation.

### 3.7 Prioritized genes were associated with immune infiltration and cellular state changes

To explore the biological context of the prioritized genes, we examined their associations with inferred immune-cell composition, simulated perturbation effects, and monocyte state transitions. Immune-cell deconvolution identified nominal between-group differences in M0 macrophages, neutrophils, and activated memory CD4T cells; however, none remained significant after Benjamini–Hochberg correction across the 22 immune-cell types (Figure 6A). These comparisons were therefore interpreted as exploratory. Correlation analysis showed that *ANPEP* and *CCL20* were positively associated with neutrophil fractions after multiple-testing correction, whereas *CD82* was negatively associated with activated memory CD4 T-cell fractions (Figure 6B). These associations provided immune-context information but did not establish direct cellular interactions. Additional supplementary analyses showed sample-level immune-cell composition, the full pattern of candidate gene–immune-cell associations, and correlations among inferred immune-cell populations (Supplementary Figures S7A–C). Only associations remaining significant after Benjamini–Hochberg correction were marked in the corresponding association plots.

**Figure 6:**
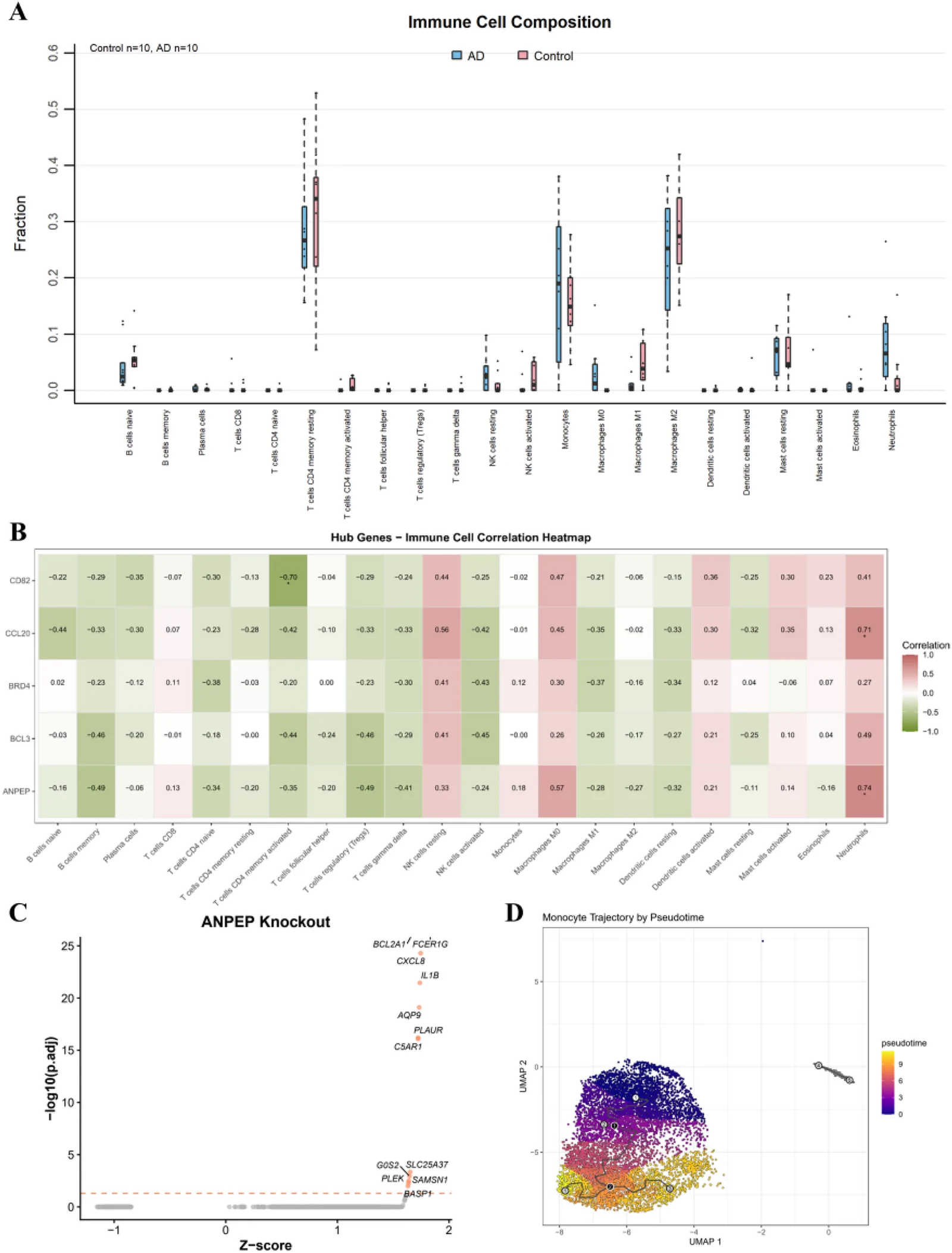
Immune deconvolution, simulated perturbation, and monocyte trajectory analyses. **(A)** CIBERSORT-estimated immune-cell fractions in the discovery cohort, comprising 10 control and 10 aortic dissection samples. Between-group comparisons were performed using two-sided Wilcoxon rank-sum tests. Nominal *P* values were 0.0149 for M0 macrophages, 0.0227 for neutrophils, and 0.0298 for activated memory CD4 T cells. None remained significant after Benjamini–Hochberg correction across 22 immune-cell types (adjusted *P* = 0.218 for all three comparisons); these differences were therefore interpreted as exploratory. **(B)** Spearman correlations between candidate-gene expression and inferred immune-cell fractions. *P* values were adjusted across 110 gene–immune-cell comparisons using the Benjamini–Hochberg method. Associations remaining significant included *ANPEP* with neutrophils (*ρ* = 0.741, adjusted *P* = 0.0207), *CCL20* with neutrophils (*ρ* = 0.706, adjusted *P* = 0.0245), and *CD82* with activated memory CD4 T cells (*ρ* = −0.695, adjusted *P* = 0.0245). **(C)** In silico simulated *ANPEP* knockout analysis showing genes predicted to be transcriptionally perturbed. **(D)** Monocyte trajectory analysis showing cellular states along the inferred pseudotime axis. Panels C and D are hypothesis-generating computational analyses.

Simulated *ANPEP* knockout predicted marked transcriptional perturbations in several myeloid- and inflammation-associated genes, including *FCER1G*, *BCL2A1*, *CXCL8*, *IL1B*, *AQP9*, *PLAUR*, and *C5AR1* (Figure 6C). Simulated knockout analyses of *BCL3*, *BRD4*, *CCL20*, and *CD82* produced distinct gene-specific perturbation patterns (Supplementary Figure S8). Because these analyses represent in silico network perturbations rather than experimental knockouts, the findings were considered hypothesis-generating and require functional validation.

Monocyte trajectory analysis revealed heterogeneous cellular states distributed along the inferred pseudotime axis (Figure 6D). Additional analyses showed the distribution of monocytes according to disease group and pseudotime, while *ANPEP*, *CCL20*, and *CD82* exhibited distinct expression patterns along the trajectory (Supplementary Figure S9). These findings provided exploratory evidence that the expression of the prioritized genes may vary across monocyte-associated cellular states but did not establish causal or temporal relationships.

### 3.8 Exploratory compound screening identified a predicted ANPEP–SB-216763 pair

To extend the gene-prioritization results into experimentally testable molecular hypotheses, we performed transcriptomic compound screening followed by molecular docking of selected compounds against the prioritized candidate targets. Transcriptomic compound screening generated a ranked list of compounds predicted to modulate the disease-associated expression signature. Based on screening rank, biological plausibility, docking feasibility, and structural availability, AACOCF3, ruxolitinib, ML320, STK-249718, and SB-216763 were selected for molecular docking (Figure 7A). Each compound was docked against ANPEP, BCL3, BRD4, CCL20, and CD82.

**Figure 7:**
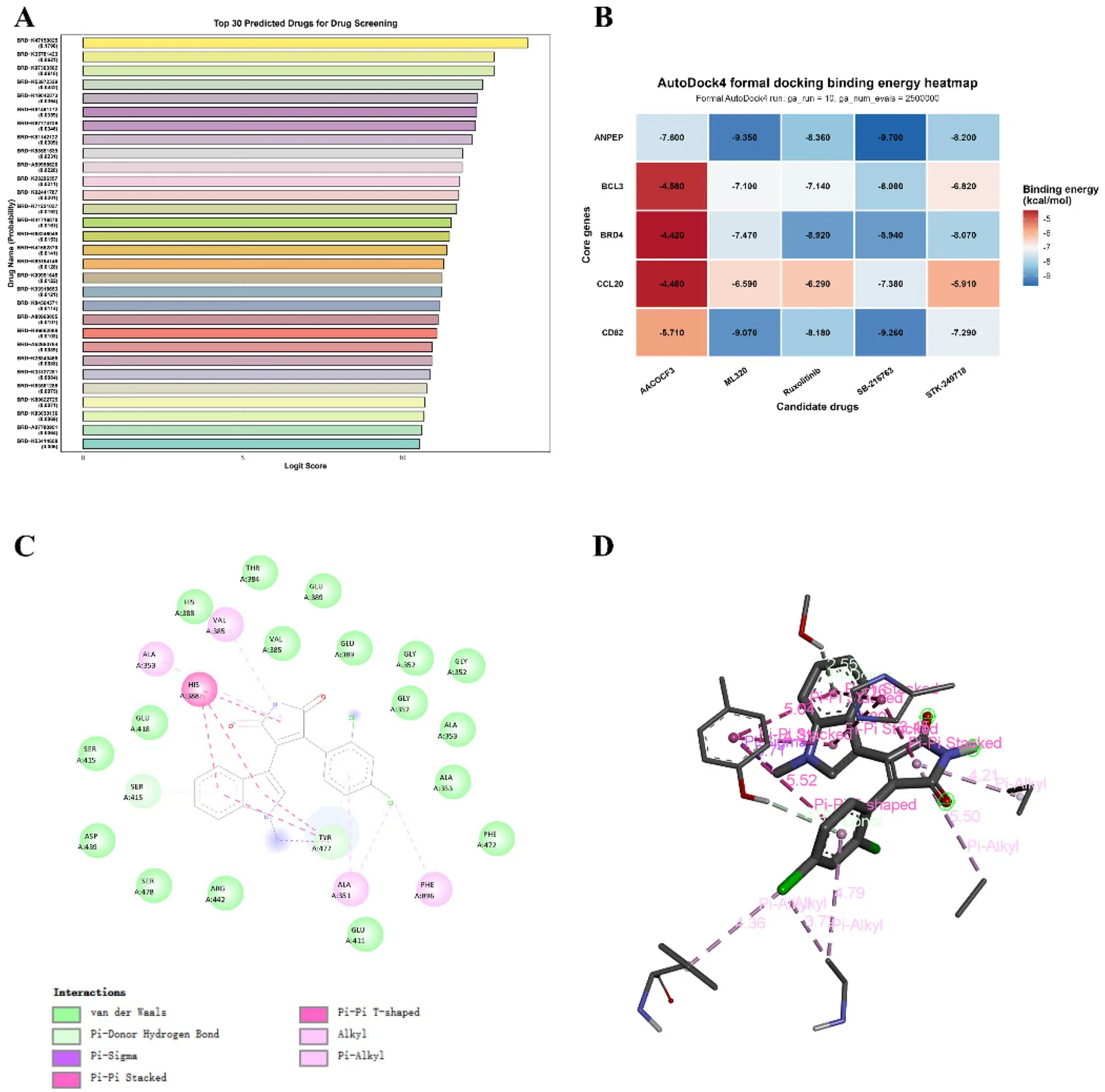
Exploratory compound screening and molecular docking. **(A)** Transcriptomic compound-screening analysis ranking compounds predicted to modulate the disease-associated transcriptional signature. **(B)** Heatmap of predicted docking energies between selected compounds and prioritized molecular candidates. **(C)** Two-dimensional interaction map of the predicted ANPEP–SB-216763 binding mode. **(D)** Three-dimensional visualization of SB-216763 accommodated within the predicted ANPEP binding pocket. The heatmap reports the minimum formal AutoDock4 score across 10 runs for each tested pair. ANPEP–SB-216763 had the lowest computed score among the 25 supplied pairs (−9.70 kcal/mol). Docking results are hypothesis-generating and require experimental validation; they do not establish biochemical binding.

Among the 25 tested compound–target pairs in this docking setup, ANPEP–SB-216763 yielded the lowest computed docking score (−9.70 kcal/mol). This result was retained as an exploratory docking hypothesis and does not establish biochemical binding, target engagement, or therapeutic activity (Figure 7B). Two-dimensional interaction mapping illustrated predicted non-covalent contacts between ANPEP and SB-216763 (Figure 7C). Three-dimensional visualization further suggested that SB-216763 could be accommodated within the predicted ANPEP binding pocket (Figure 7D). Because *ANPEP* was supported by multiple transcriptomic and cell-context evidence layers, the predicted ANPEP–SB-216763 pair was retained as an exploratory docking candidate. No biochemical binding, target-engagement, or functional assay was performed; therefore, the prediction requires biochemical and cell-based validation.

### 3.9 Spatial transcriptomics provided descriptive context for vascular microenvironmental heterogeneity

Exploratory analysis of GSE241870 was performed to provide descriptive tissue-context information regarding vascular microenvironmental heterogeneity. Sample-level module-score analysis showed variation in smooth muscle cell contractile, extracellular matrix-remodeling, endothelial, and macrophage-like programs across the spatial transcriptomic samples (Figure 8A). Dominant spatial niche analysis further illustrated differences in the relative representation of vascular and microenvironmental states across the five samples (Figure 8B).

**Figure 8:**
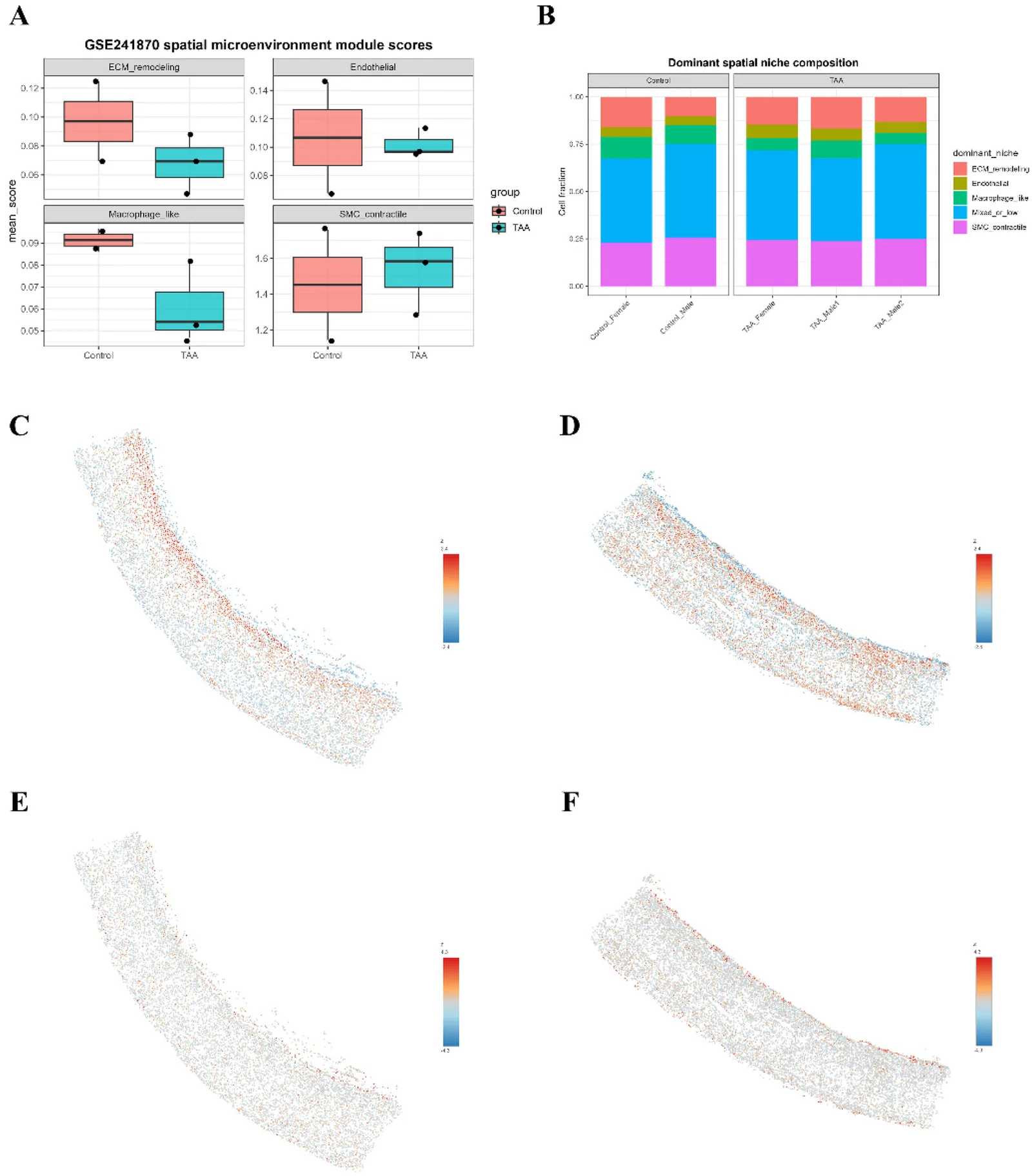
Spatial characterization of the vascular microenvironment. **(A)** Sample-level module scores for smooth muscle cell contractile, extracellular matrix-remodeling, endothelial, and macrophage-like programs in GSE241870. Each point represents one spatial transcriptomic sample; the dataset comprised two control and three thoracic aortic aneurysm samples. Boxplots show the median and interquartile range. Because of the small sample size and the non-aortic-dissection disease context, the group differences are presented descriptively without inferential statistical testing. **(B)** Dominant spatial niche composition across the five samples. **(C,D)** Spatial distribution of smooth muscle cell contractile scores in representative control and thoracic aortic aneurysm sections. **(E,F)** Spatial distribution of extracellular matrix remodeling scores. This dataset was used as supplementary microenvironmental evidence rather than direct validation of the prioritized genes.

Spatial mapping demonstrated regional heterogeneity in smooth muscle cell contractile and extracellular matrix-remodeling scores in representative control and thoracic aortic aneurysm sections (Figures 8C–F). Additional endothelial and macrophage-like module-score maps are presented in Supplementary Figure S10. Because GSE241870 represented thoracic aortic aneurysm rather than aortic dissection and did not contain the prioritized genes, these findings were interpreted only as supplementary tissue-context evidence and not as direct validation.

## 4 Discussion

This study integrated bulk and single-cell transcriptomics with genetic risk information and cell-type-specific co-expression analysis to prioritize monocyte-associated molecular candidates in aortic dissection. The principal finding was the convergence of bulk differential expression, GWAS-informed single-cell evidence, monocyte co-expression structure, an exploratory secondary-cohort assessment, and preliminary THP-1 transcriptional assessment on ANPEP. Because GSE52093 contributed to the historical integrated evidence matrix and was subsequently reused for cohort-level assessment, it should not be considered a fully independent validation cohort; given its small size, it is interpreted as supportive cross-cohort evidence rather than clinical validation. *CCL20* and *CD82* provided supporting but less consistent evidence. Immune-context, trajectory, simulated-perturbation, compound-screening, and spatial analyses were exploratory and were not used to establish causal function or therapeutic activity.

The contribution of monocyte/macrophage activation to aortic wall injury has been recognized previously. The added value of the present study is not the proposal of monocyte involvement alone, but the convergence of bulk differential expression, genetic risk-informed single-cell evidence, cell-type-specific co-expression structure, exploratory secondary-cohort assessment, and preliminary author-generated transcriptional assessment on a focused set of inflammatory candidates. This convergence prioritized *ANPEP* as a comparatively consistent candidate while also demonstrating the cohort sensitivity of *CCL20*.

The prominence of monocytes is biologically plausible because circulating monocytes and macrophage-lineage cells can translate systemic inflammatory cues into local vascular injury (14). These cells may promote cytokine release, protease activity, oxidative stress, and extracellular matrix turnover, while also influencing the smooth muscle cell phenotype (8, 10, 14). In the present analysis, the monocyte blue module overlapped both upregulated bulk genes and genetically informed scPagwas candidates. Immune deconvolution further linked *ANPEP* to neutrophil fractions after multiple-testing correction, whereas the association with M0 macrophages remained nominal. Although these associations cannot establish directionality or causation, their convergence across tissue-level, cell-level, and genetic evidence makes the monocyte signal less likely to represent an isolated dataset artifact. The direct single-cell audit further showed the highest ANPEP expression and detection fraction in the existing broad Monocyte annotation, with substantial expression in Neutrophils; this supports a monocyte-associated but non-exclusive pattern. Because the available labels were broad, the analysis did not establish a classical or non-classical monocyte or macrophage subtype, and no inflammatory macrophage signature correlation was inferred.

Smooth muscle cells remained important even though the final candidate molecular signature was derived primarily from the monocyte-centered intersection. Medial smooth muscle cells maintain contractile tone, extracellular matrix organization, and mechanical integrity of the aortic wall (8, 15, 16). The broad overlap between the smooth muscle cell turquoise module and downregulated disease genes may reflect impaired contractile programs or altered matrix homeostasis. Enrichment of focal adhesion, integrin, hypoxia, PI3K-Akt, MAPK, and shear stress-related pathways is consistent with this interpretation. Accordingly, the immune and smooth muscle cell findings should be viewed as complementary components of the same disease microenvironment rather than competing explanations.

Within this immune-vascular framework, the three prioritized genes may capture different facets of disease biology. ANPEP is a membrane-associated ectoenzyme involved in peptide processing, leukocyte behavior, cell adhesion, and tissue remodeling (24). Mechanistically, experimental studies suggest that CD13/ANPEP can modulate myeloid-cell activation, inflammatory signaling, and monocyte–endothelial adhesion, including CD11b/CD18 activation and cytokine release after CD13 engagement (25, 26). CD13 has also been linked to endothelial-cell adhesion, migration, and MMP-related extracellular-matrix remodeling, providing a plausible route by which ANPEP-expressing myeloid cells could indirectly influence vascular smooth muscle cell behavior and extracellular-matrix homeostasis (27, 28). However, these mechanisms were not directly tested in the present study; our findings therefore remain hypothesis-generating rather than evidence of a causal ANPEP-mediated pathway in aortic dissection.

Its presence in the monocyte module, upregulation in bulk tissue, higher expression in the secondary cohort, and association with immune estimates make it the most coherent cross-layer candidate in this analysis. CCL20 provides a chemokine-related link to leukocyte recruitment and inflammatory communication (29). However, its weaker cross-cohort expression consistency indicates that it may be more sensitive to cohort composition or disease stage. CD82, a tetraspanin involved in membrane organization and cell-cell interactions, showed higher expression in the secondary cohort but has a less established role in aortic dissection (30, 31). The controlled three-gene model evaluated joint dataset-level discrimination rather than gene-specific diagnostic performance. These differences argue against treating all three genes as mechanistically interchangeable. The preliminary ddPCR experiment further showed that Ang II stimulation increased *CCL20* and *ANPEP* transcript abundance in THP-1 macrophage-like cells. The stronger induction of *CCL20* was consistent with its role as an inflammatory chemokine, whereas the more moderate increase in *ANPEP* supported its transcriptional responsiveness in this model. However, these results did not establish the causal involvement of either gene and should not be considered equivalent to validation in primary monocytes, patient blood, or aortic tissue.

These results should be interpreted cautiously. The controlled rerun yielded an AUC of 0.85 in nested leave-one-out evaluation of the 20-sample discovery cohort and an AUC of 1.00 in the 12-sample secondary cohort. The latter result is imprecise and exploratory and should not be interpreted as fully independent validation because GSE52093 contributed to the historical integrated evidence matrix. Although fold-specific preprocessing and nested tuning reduce within-fold leakage, they cannot remove optimism introduced by upstream candidate selection. The small public cohorts, tissue and platform differences, and limited case composition may also inflate apparent discrimination. These results should not be interpreted as evidence of clinical diagnostic utility or biomarker performance. Larger prospectively collected cohorts, including independent blood-based samples and comparisons with other acute aortic syndromes, are required before clinical interpretation.

The compound-screening analysis extended this prioritization into experimentally testable molecular hypotheses rather than treatment recommendations. In the formal docking setup, ANPEP–SB-216763 had the lowest computed score among the 25 tested pairs (−9.70 kcal/mol), and the interaction maps suggested that the compound could be accommodated within a local binding pocket through several non-bond contacts. This comparison is protocol-specific and hypothesis-generating; it does not establish direct binding, target modulation, cellular efficacy, or safety. Docking scores, however, are sensitive to receptor preparation, pocket definition, ligand protonation, and the scoring function (32). They do not demonstrate direct biochemical binding, target modulation, cellular efficacy, or safety. The ANPEP–SB-216763 pair should therefore be regarded as an exploratory docking candidate for future binding and cell-based assays.

The spatial analysis added a complementary tissue-context layer but had a deliberately limited role. Because the targeted panel did not cover the complete core-gene set, it was used to examine smooth muscle cell, endothelial, macrophage-like, and extracellular matrix-related features. Regional variation in these scores supports microenvironmental heterogeneity within the diseased ascending aorta and is compatible with the bulk and single-cell findings (23). Nevertheless, the spatial dataset represents a different aortic disease context and cannot substitute for spatial profiling of a dedicated aortic dissection cohort. Future studies using transcriptome-wide spatial technologies could directly test whether ANPEP-positive monocyte/macrophage niches localize to regions of smooth muscle cell loss or active matrix remodeling.

Taken together, several strengths increase the interpretability of the study. Independent evidence layers were combined rather than relying on a single differential-expression list; cell-type-specific modules were evaluated, and selected genes underwent exploratory secondary-cohort assessment. The analysis also preserved a distinction between prioritized molecular candidates, auxiliary mechanistic genes, and exploratory compound-screening hypotheses. Important limitations remain. All datasets were retrospective and publicly available; sample sizes were limited, and platform or tissue heterogeneity may introduce residual bias. The historical multi-algorithm workflow also involved feature selection before resampling and did not use nested cross-validation; those outputs were therefore not retained as confirmatory diagnostic evidence. GSE52093 was a distinct secondary cohort but was not an untouched validation set because it contributed to retrospective candidate/model assessment. The study also lacks independent blood-based validation from patients with aortic dissection. The controlled nested-resampling rerun addresses within-fold preprocessing and tuning leakage, but upstream candidate selection and the small cohort sizes still limit performance interpretation. The scPagwas, hdWGCNA, immune deconvolution, trajectory, simulated knockout, and docking analyses are inferential. The THP-1 ddPCR experiment provided only preliminary transcriptional-level support and does not replace validation in primary macrophages or human aortic dissection tissues. The THP-1 experiment did not constitute replication in an independent patient cohort and did not test causal function. No gene-silencing, protein-level, biochemical binding, or disease-relevant functional assay was performed. Consequently, the candidate genes and compound–target prediction require validation in primary cells, independent patient samples, and functional perturbation experiments. The single-cell ANPEP audit used existing broad annotations and descriptive donor summaries; it did not resolve specific monocyte/macrophage subtypes or test a prespecified inflammatory macrophage signature.

## 5 Conclusion

Integration of bulk transcriptomic, single-cell, genetic-risk, and cell-type-specific co-expression evidence identified a monocyte-associated molecular program in aortic dissection. ANPEP was prioritized as a comparatively consistent monocyte-associated molecular candidate within the integrated discovery evidence and supportive secondary-cohort assessment, together with preliminary THP-1 ddPCR evidence. CCL20 and CD82 were retained as supporting candidates with distinct but less complete evidence profiles. These findings prioritize experimentally testable hypotheses and do not establish independent clinical validation, causal function, diagnostic utility, or therapeutic activity. Further evaluation in larger independent cohorts, including blood-based patient samples, and functional perturbation models is required.

## Data Availability

The publicly available datasets analyzed in this study are accessible through the Gene Expression Omnibus under accession numbers GSE153434, GSE52093, GSE189795, and GSE241870. GWAS summary statistics were obtained from FinnGen. The author-generated ddPCR data, processed data, and analysis scripts supporting the findings are available from the corresponding author, Yongbin Shi (shi19870518@126.com), upon reasonable request.
