# Supplementary material for: Genetic-risk-informed integration of bulk and single-cell transcriptomes prioritizes ANPEP as a monocyte-associated candidate in aortic dissection
Source: Front Cardiovasc Med. 2026 Oct 7;13:1965670. doi: 10.3389/fcvm.2026.1965670 (PMC13682983; doi:10.3389/fcvm.2026.1965670)
Supplement: Supplementary file 1 [file Datasheet1.pdf]

Supplementary Material

1 Supplementary Figures

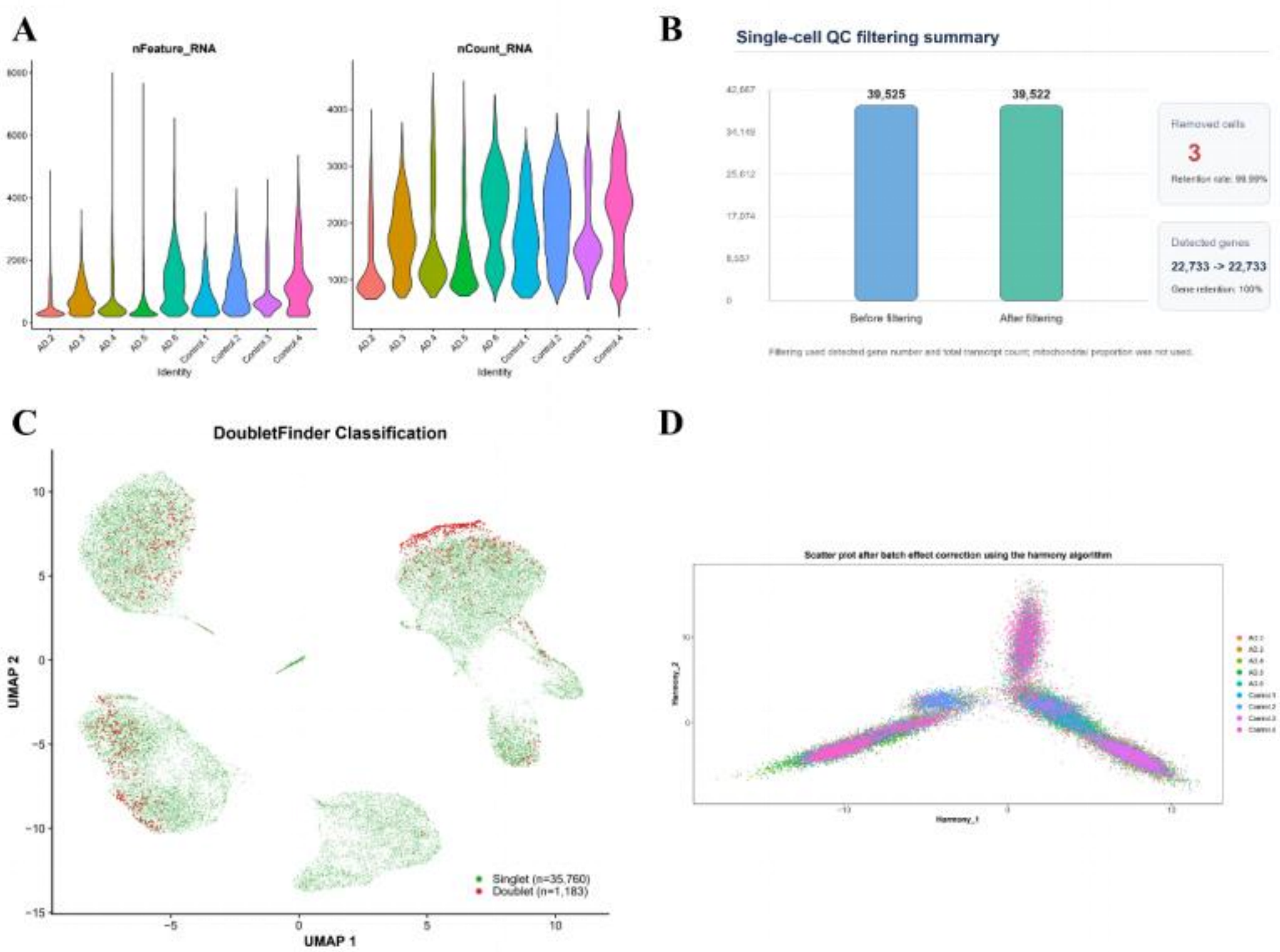

Supplementary Figure S1. Single-cell quality-control analysis. (A) Distributions of detected gene numbers and total transcript counts before quality-control filtering. (B) Summary of cell and gene retention after filtering, showing the numbers of cells and detected genes retained in the processed dataset. Mitochondrial transcript proportion was not used because canonical mitochondrial genes were unavailable in the processed matrix. (C) DoubletFinder-based classification of singlets and potential doublets. (D) Low-dimensional representation following Harmony batch correction.

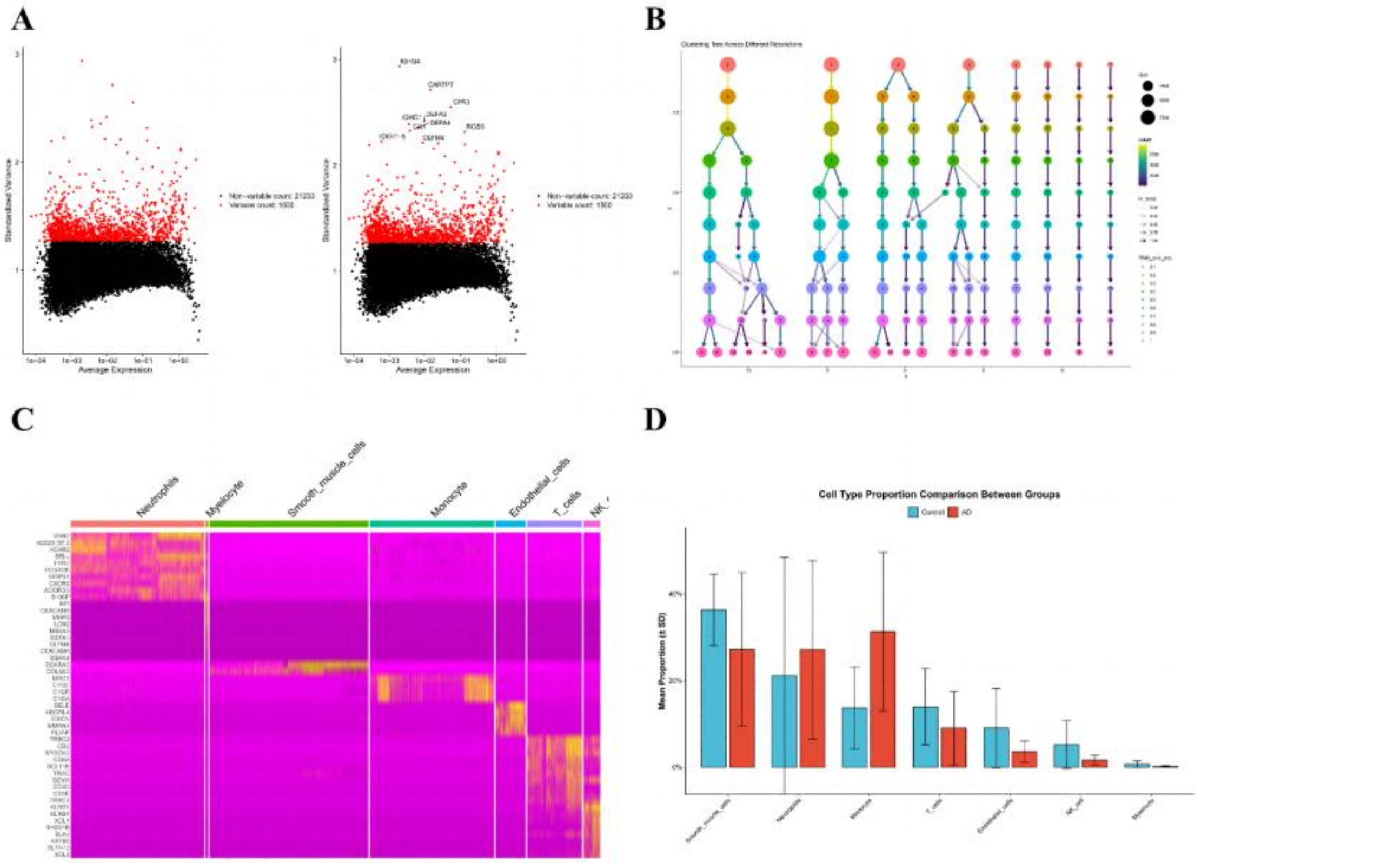

Supplementary Figure S2. Feature selection and cell composition. (A) Highly variable feature selection was used for downstream single-cell dimensionality reduction and clustering. (B) Clustree analysis supporting clustering-resolution assessment. (C) Heatmap of representative marker genes supporting cell-type annotation. (D) Cell-type proportion comparison across disease groups. Cell-type proportions were calculated at the sample level using four control and five aortic dissection samples. The comparisons are presented descriptively because of the limited number of biological samples.

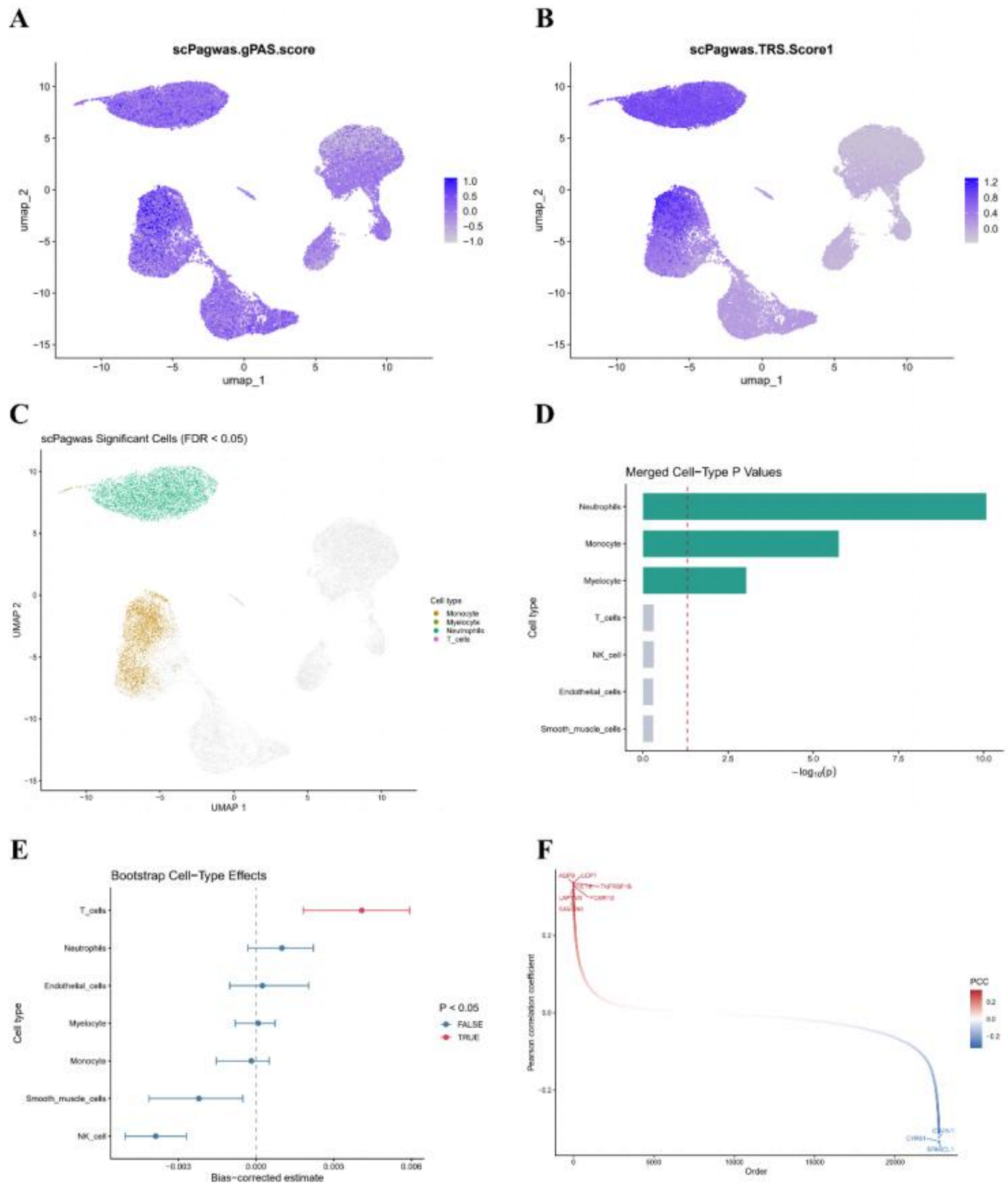

Supplementary Figure S3. Extended scPagwas results. (A) Distribution of scPagwas genetic pathway activity scores across the single-cell landscape. (B) Distribution of trait-relevance scores across cells. (C) UMAP visualization of significant risk-associated cells. (D) Cell-type association statistics from scPagwas analysis. (E) Bootstrap estimates supporting genetically associated cell-type prioritization. (F) Distribution of gene-level Pearson correlation coefficients used to support gene prioritization.

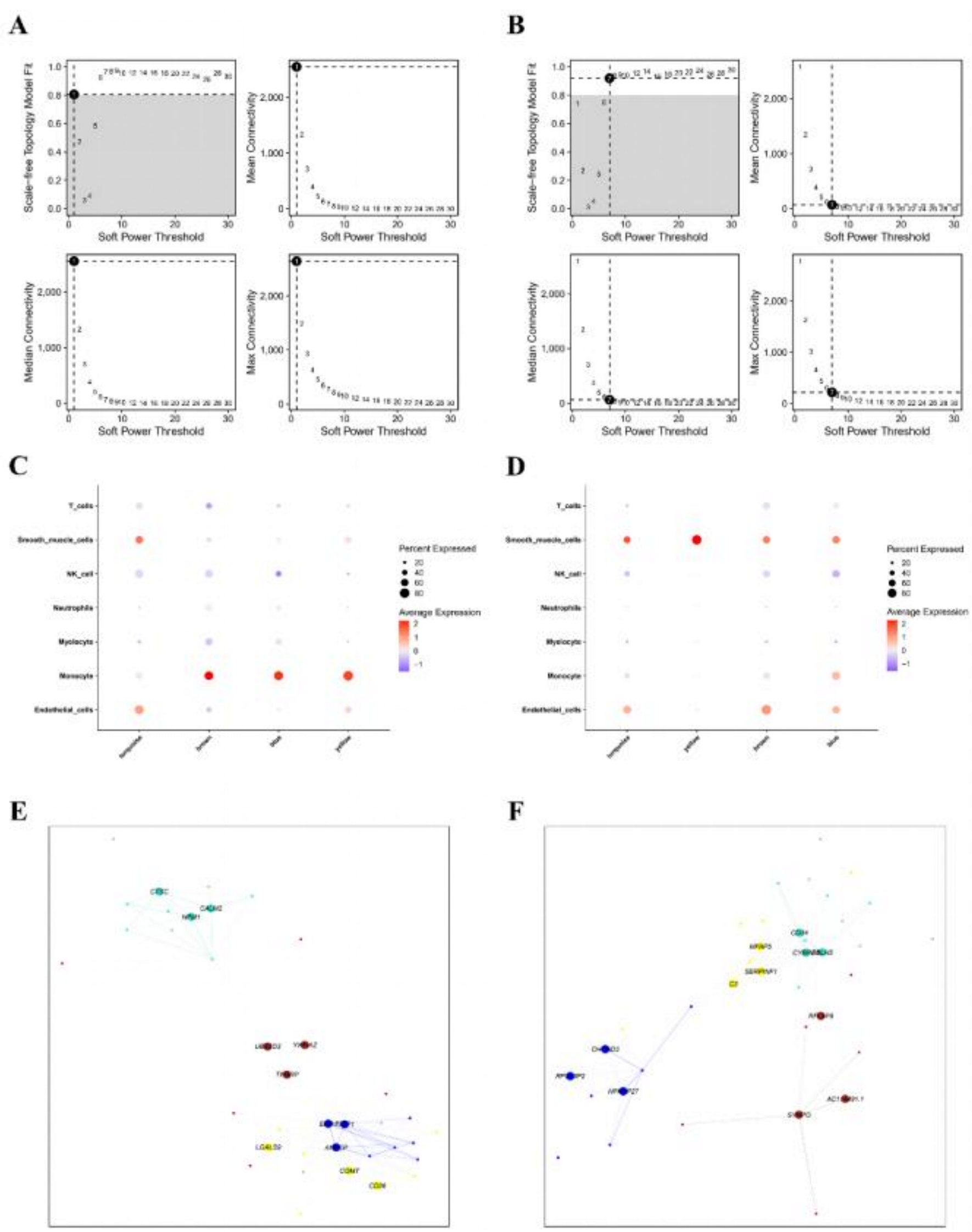

Supplementary Figure S4. hdWGCNA diagnostics. (A) and (B) Soft-threshold selection for monocyte and smooth muscle cell hdWGCNA network construction. (C) and (D) Module–cell-type association plots for monocyte and smooth muscle cell co-expression modules. (E) and (F) Hub-gene network visualizations for selected monocyte and smooth muscle cell modules.

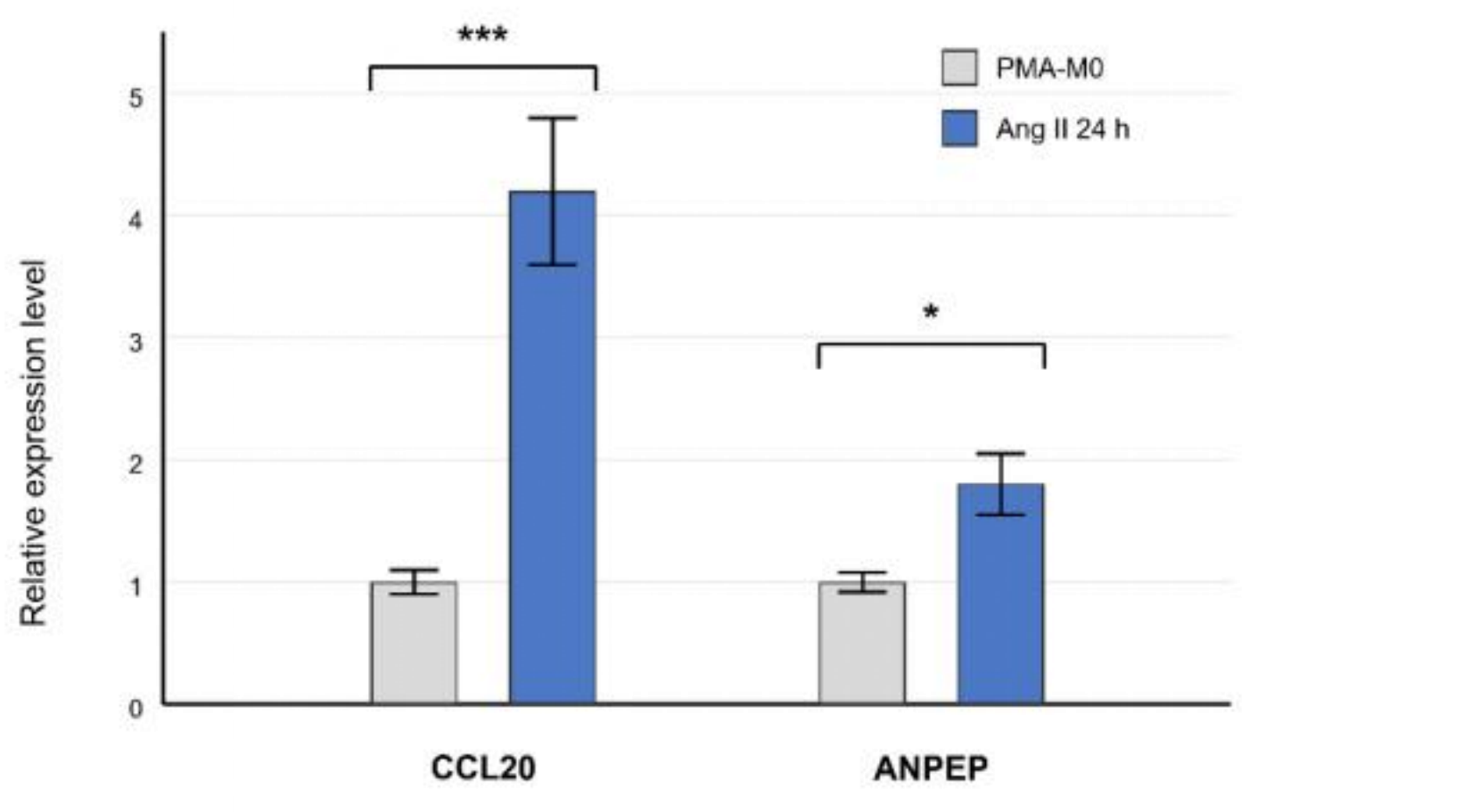

Supplementary Figure S5. Preliminary ddPCR assessment in Ang II-stimulated THP-1 macrophage-like cells. *CCL20* and *ANPEP* transcript abundance was measured in PMA-differentiated THP-1 macrophage-like cells under control and Ang II-treated conditions. Five independent biological experiments were performed, with control and Ang II-treated cells derived from the same culture preparation in each experiment. Data are presented as mean ± SD. Comparisons were performed using a two-sided paired t-test. *P < 0.05, ***P < 0.001.

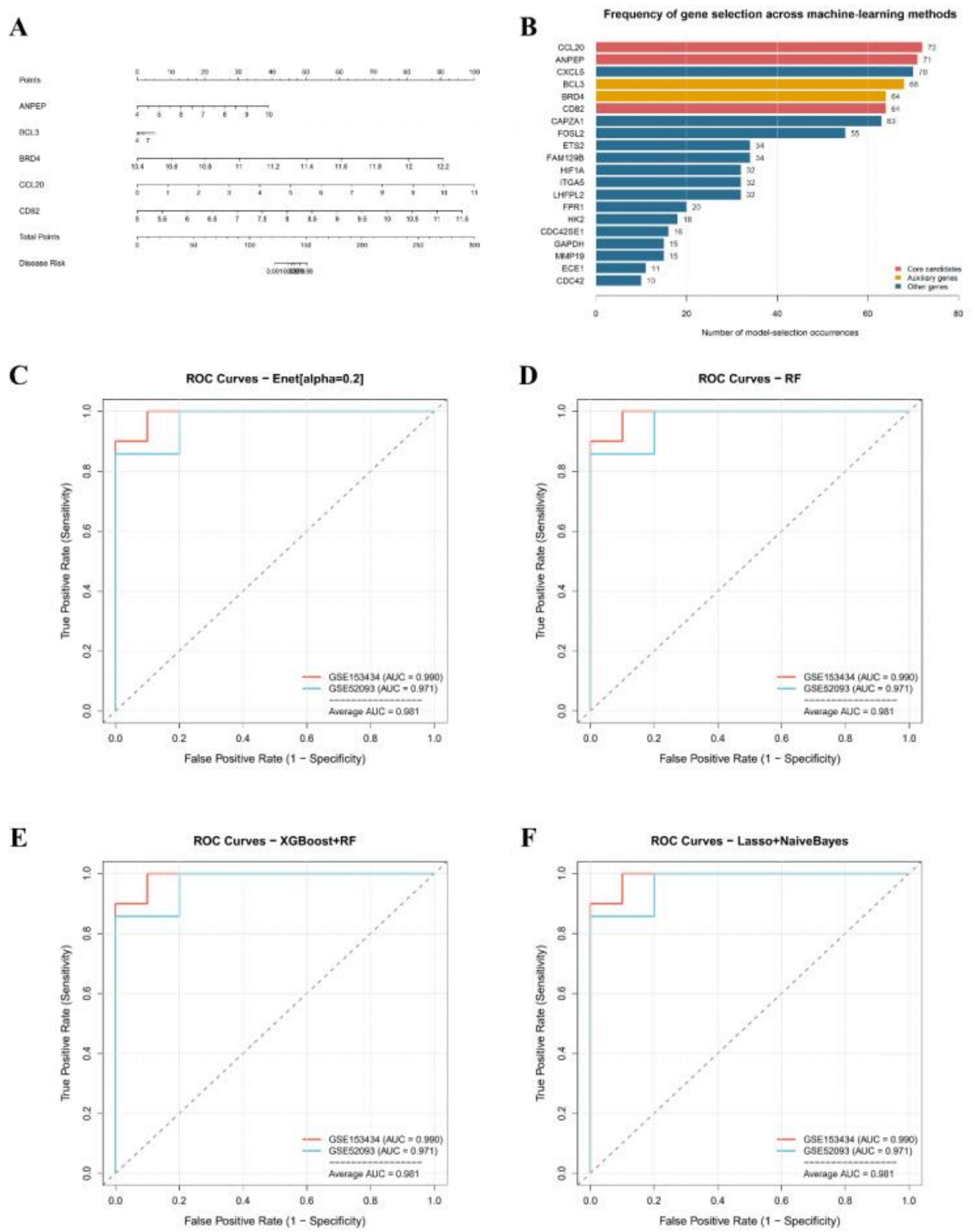

Supplementary Figure S6. Historical exploratory machine-learning outputs retained for transparency only. These panels were generated by the original workflow and are not used to support confirmatory discrimination claims because feature selection occurred before resampling, nested cross-validation was not performed, some model-by-cohort units were incomplete, and saved-score AUCs were inconsistent with the historical AUC table. The revised primary analysis is presented in Figure 5.

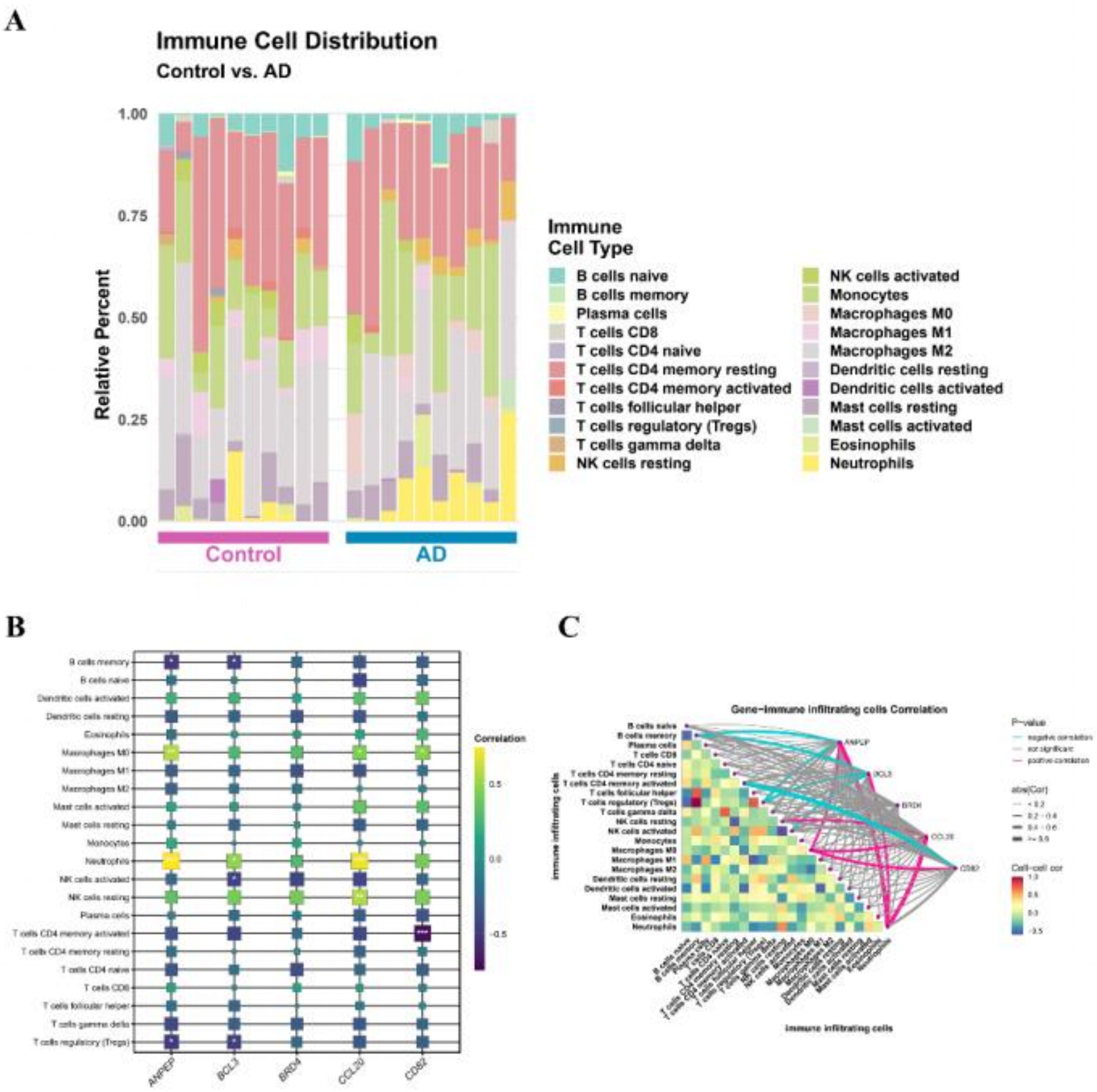

Supplementary Figure S7. Immune microenvironment analyses. (A) Estimated immune-cell composition across individual samples inferred using CIBERSORT. (B) Bubble plot summarizing associations between candidate genes and immune-cell fractions. (C) Correlation matrix showing relationships among inferred immune-cell populations and candidate-gene associations. These analyses were interpreted as exploratory immune-context evidence. Between-group immune-cell comparisons were performed using two-sided Wilcoxon rank-sum tests, with Benjamini–Hochberg correction across 22 cell types. Gene–immune-cell associations were assessed using Spearman correlation, with Benjamini–Hochberg correction across 110 comparisons. Only associations remaining significant after Benjamini–Hochberg correction are marked in the figure.

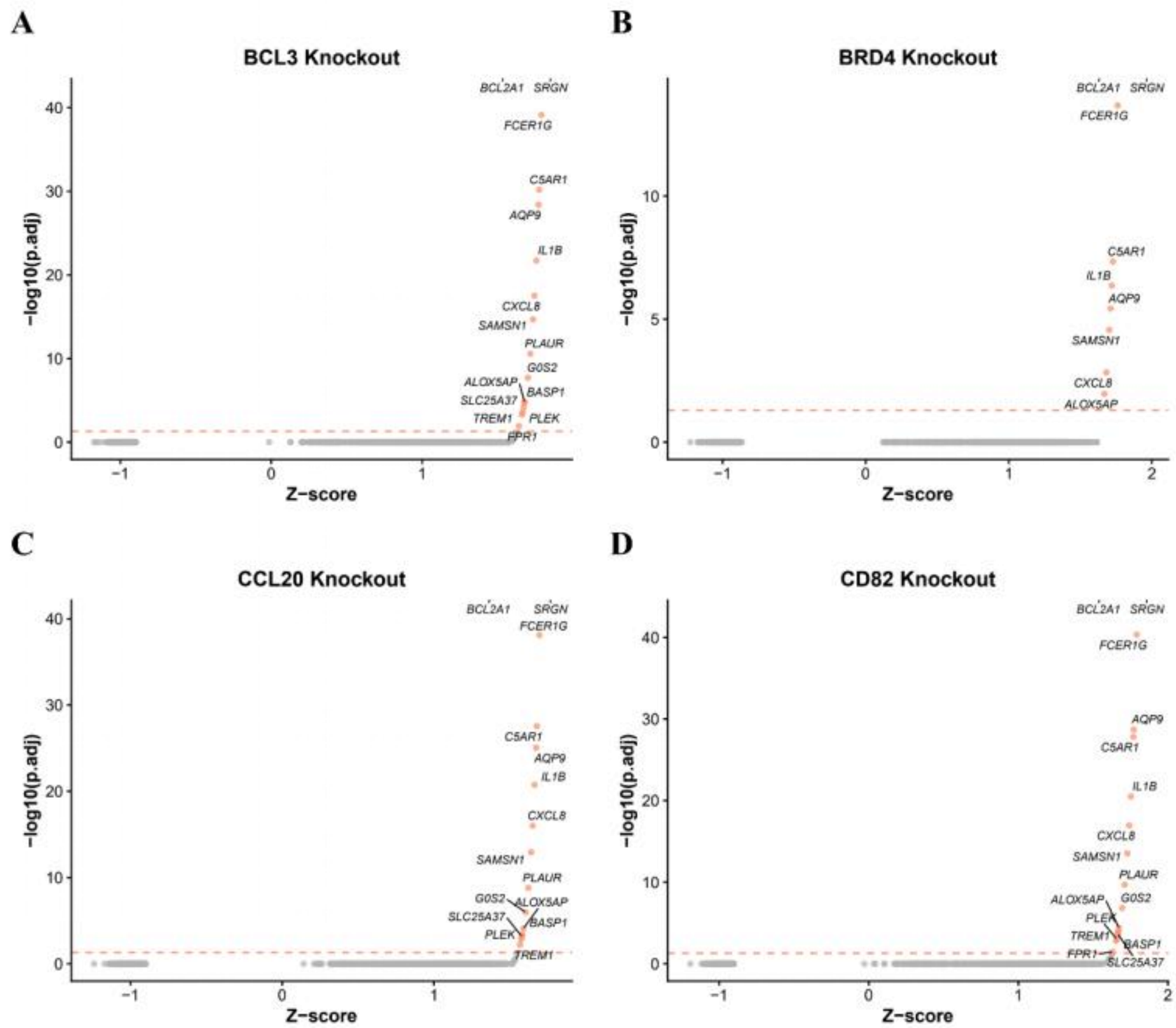

Supplementary Figure S8. Simulated knockout of candidate genes. (A–D) In silico simulated knockout analyses of *BCL3*, *BRD4*, *CCL20*, and *CD82*, respectively. Differentially perturbed genes are shown for each simulated knockout model. These computational perturbation analyses are hypothesis-generating and do not substitute for experimental gene knockout validation.

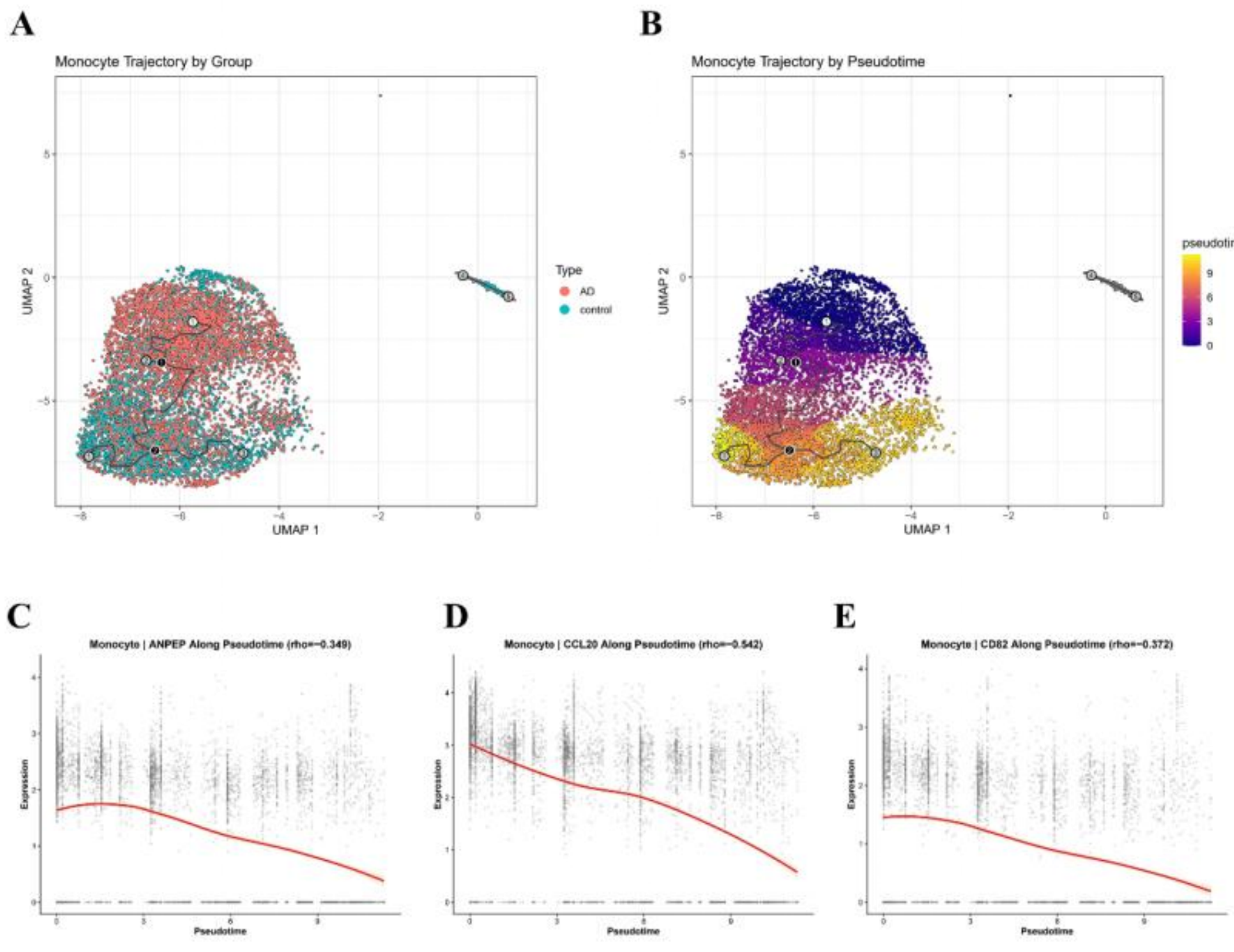

Supplementary Figure S9. Monocyte trajectory and gene dynamics. (A) Monocyte trajectory stratified by disease group. (B) Monocyte trajectory colored by inferred pseudotime. (C–E) Expression dynamics of *ANPEP*, *CCL20*, and *CD82* along the monocyte pseudotime trajectory. These analyses provide exploratory evidence of gene-expression variation across monocyte-associated cellular states.

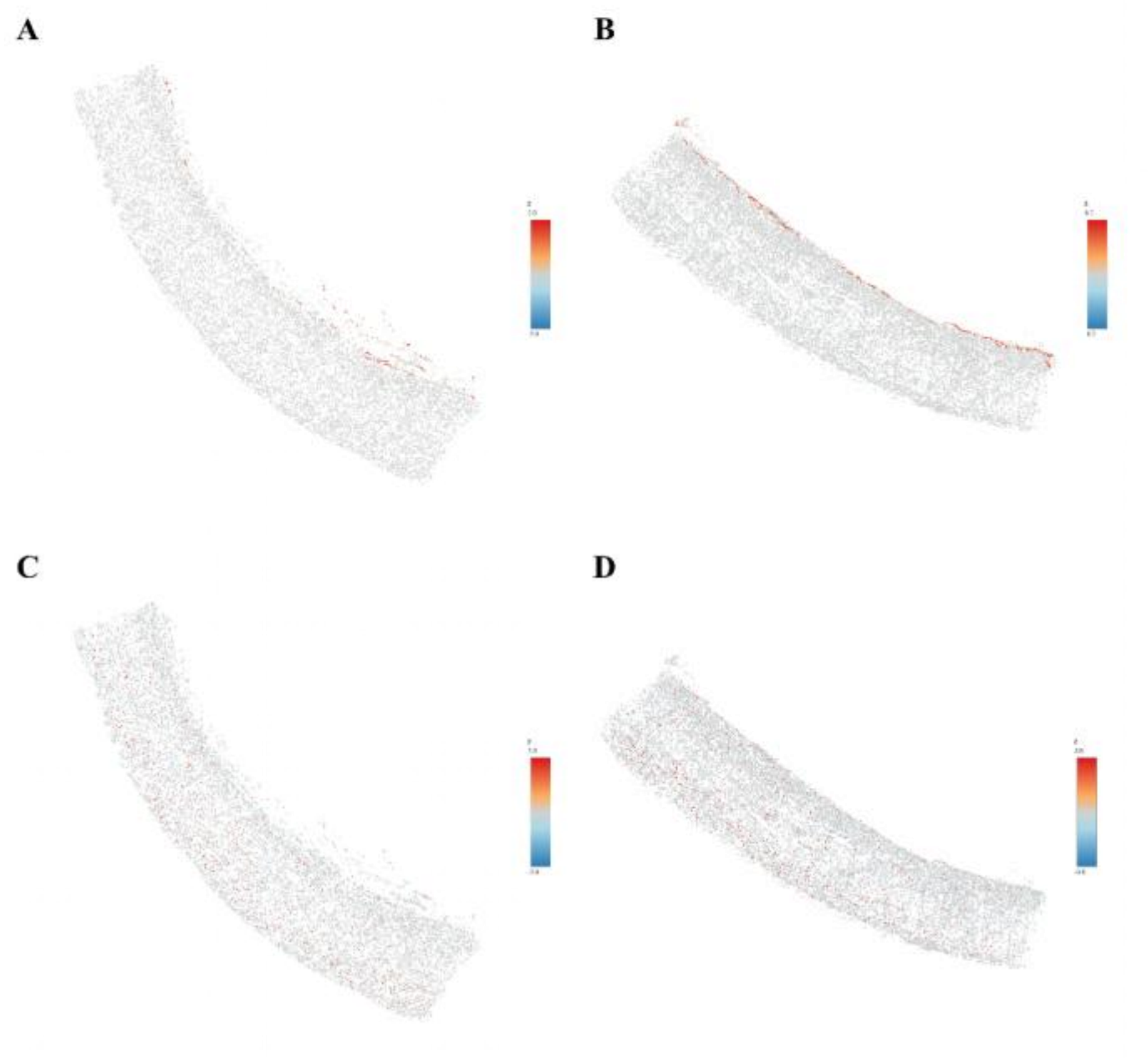

Supplementary Figure S10. Spatial microenvironment maps. (A, B) Spatial distribution of endothelial module scores in representative control and thoracic aortic aneurysm sections. (C, D) Spatial distribution of macrophage-like module scores in representative control and thoracic aortic aneurysm sections. These maps complement the main spatial analysis by showing additional vascular and immune microenvironmental features.

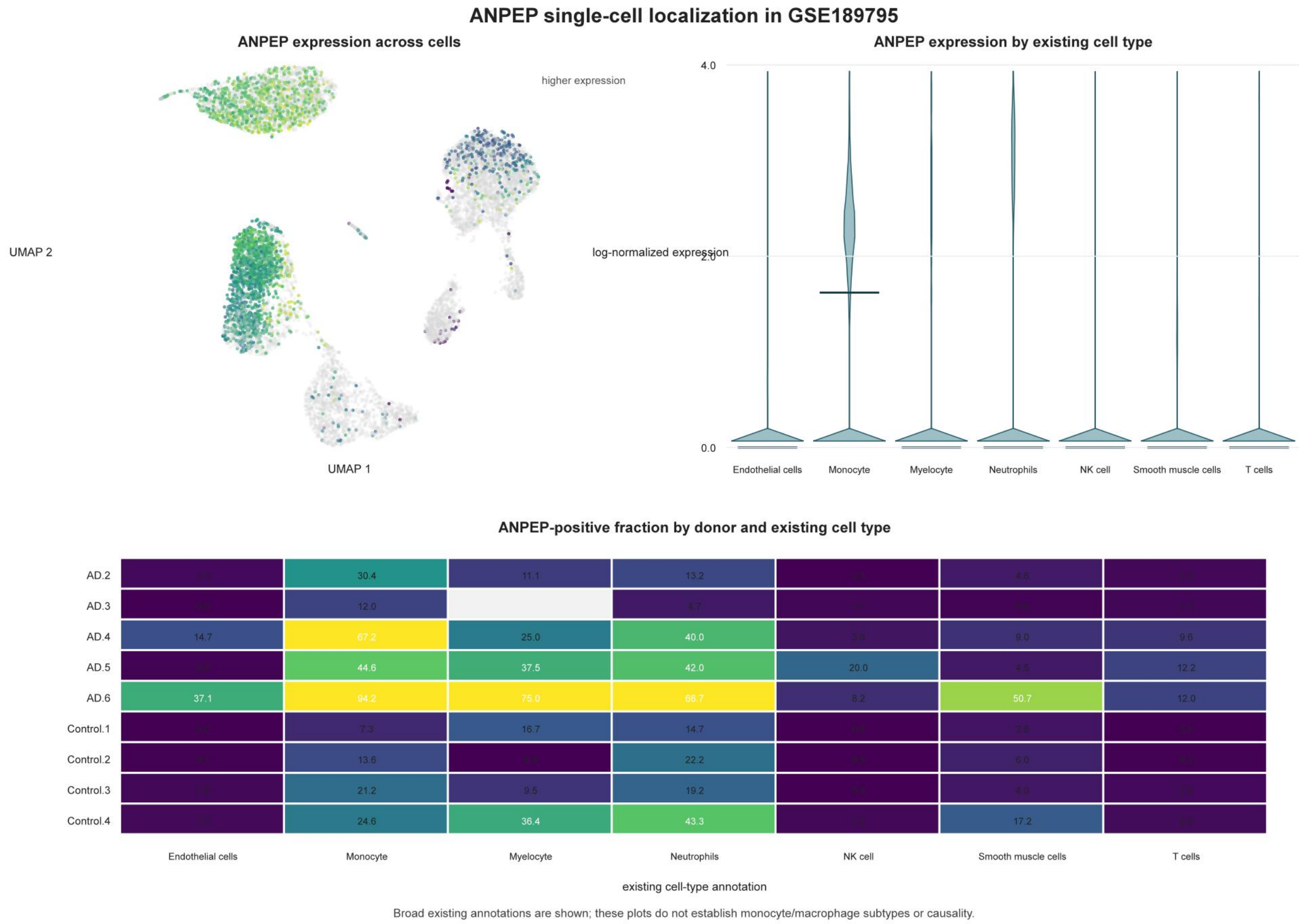

Supplementary Figure S11. Single-cell localization of ANPEP in the GSE189795 dataset. (A) UMAP feature plot showing log-normalized ANPEP expression across 35,760 cells from nine donors. (B) ANPEP expression distribution across the existing broad cell-type annotations. Monocytes showed the highest mean expression (1.236) and proportion of ANPEP-positive cells (52.6%), followed by neutrophils (0.832 and 27.0%, respectively). (C) Donor-by-cell-type heatmap of the proportion of ANPEP-positive cells. These descriptive analyses support a monocyte-associated but non-exclusive pattern; the existing annotations do not resolve specific monocyte/macrophage subtypes or establish an inflammatory macrophage signature.

2 Supplementary Tables

Supplementary Table S1. Summary of datasets and study cohorts

Dataset	Data type	Tissue/source	Sample size	Role in study	PMID
**GSE153434**	Bulk RNA-seq	Ascending aortic tissue	Aortic dissection: 10; Control: 10	Discovery transcriptomic cohort	33066131
**GSE52093**	Expression microarray	Ascending aortic tissue	Aortic dissection: 7; Control: 5	Exploratory secondary-cohort assessment (not clinical validation)	24586754
**GSE189795**	Single-cell RNA-seq	Ascending aortic tissue	Aortic dissection: 5; Control: 4; 35,760 cells after QC	Single-cell cellular-context analysis	36536281
**FinnGen I9_AORTDIS_EXNONE**	GWAS summary statistics	Population-level genetic data	Summary statistics	Genetic risk-informed scPagwas analysis	36653562
**GSE241870**	Targeted spatial transcriptomics (MERSCOPE)	Ascending aortic tissue	Thoracic aortic aneurysm: 3; Control: 2	Supplementary spatial microenvironment analysis	37823268

Supplementary Table S2. Primer sequences used for ddPCR assessment

Primer information	*CCL20*	*ANPEP*	*GAPDH*
**Forward primer sequence (5′–3′)**	TGCTGTACCAAGAGTTTGCTC	GACCAAAGTAAAGCGTGGAATCG	ACAACTTTGGTATCGTGGAAGG
**Reverse primer sequence (5′–3′)**	CGCACACAGACAACTTTTTCTTT	TCTCAGCGTCACCCGGTAG	GCCATCACGCCACAGTTTC
**Amplicon size**	220 bp	75 bp	101 bp
**Source / PrimerBank ID**	PrimerBank ID: 4759076a1	PrimerBank ID: 157266299c1	PrimerBank ID: 378404907c2

Supplementary Table S3. Software, versions, and confirmed major parameters used in this study.

Analysis module	Software or package	Version	Confirmed major parameters/settings
**Computing environment**	R and Python with custom scripts	R 4.3.2; Python 3.10.13	Custom R and Python scripts; no additional environment settings reported.
**Bulk differential expression**	limma	limma 3.58.1	DEG threshold: |log2 fold change| > 0.5 and Benjamini-Hochberg-adjusted P < 0.05.
**Functional enrichment**	clusterProfiler; org.Hs.eg.db	clusterProfiler 4.10.1; org.Hs.eg.db 3.18.0	Gene Ontology and KEGG analyses; additional package-specific settings not reported.
**Single-cell preprocessing and clustering**	Seurat; SeuratObject	Seurat 5.0.1; SeuratObject 5.0.1	CreateSeuratObject: min.cells = 10 and min.features = 200; cells with nFeature_RNA ≥ 200 were retained; no fixed upper nFeature_RNA or nCount_RNA cutoffs were applied. Canonical mitochondrial genes were unavailable in the processed matrix, so percent.mt filtering was not applied. Ambient RNA was filtered per sample using decontX thresholds of Q3 + 1.5 × IQR constrained to 0.10–0.30. Doublets were evaluated per sample: scDblFinder was used for samples with <100 cells; otherwise DoubletFinder was used with PCs 1–10, pN = 0.25, pK selected by maximal BCmetric, and homotypic-adjusted nExp. Data were log-normalized with scale.factor = 10,000; 1,500 variable genes were selected using VST; 20 PCs were calculated, Harmony was run with sample_id as the grouping variable, and PCs 1–13 from the Harmony reduction were used for downstream analysis. The final clustering resolution was 0.3 after a diagnostic sweep from 0.1 to 1.0 in increments of 0.1; UMAP used Harmony PCs 1–13.
**Batch correction**	Harmony	Harmony 1.2.0	Harmony was applied after PCA with group.by = sample_id; 20 PCs were calculated and PCs 1–13 from the Harmony reduction were used for neighbors, clustering, and UMAP.

**Genetic risk-informed single-cell analysis**	scPagwas	scPagwas 1.0.0	RNA assay; hg37 genome build; seed = 123; maf_filter = 0.01; marg = 10,000; min_clustercells = 10; pathway size = 10–120; iters_celltype = 50; iters_singlecell = 25; n_topgenes = 400; n.cores = 8; run_split = FALSE; singlecell = TRUE; celltype = TRUE; seurat_return = TRUE; remove_outlier = TRUE. Reference resources were loaded from the scPagwas installation, including the KEGG gene–pathway mapping, LD structure, and hg37 block annotation. Positive PCC genes were used for monocyte integration and negative PCC genes for smooth muscle cell integration.
**Cell-type-specific co-expression analysis**	hdWGCNA; WGCNA	hdWGCNA 0.3.0; WGCNA 1.72-5	SetupForWGCNA used gene_select = fraction with fraction = 0.05. Metacells were constructed by cell_type and Type with k = 25, max_shared = 10, and ident.group = cell_type. Signed networks were used; the soft-thresholding power was selected as the smallest power with SFT.$R^2 \geq 0.8$ or, if none met this criterion, the power with the maximum SFT.R^2. The saved monocyte and smooth muscle cell analyses used powers 6 and 7, respectively, with deepSplit = 4, minModuleSize = 50, and mergeCutHeight = 0.2.
**Controlled exploratory fixed-gene discrimination analysis**	glmnet; custom R scripts	glmnet 4.1-8; R 4.3.2	ANPEP, CCL20, and CD82 fixed before resampling; L2 logistic regression; fold-specific standardization; outer leave-one-out cross-validation in GSE153434; inner three-fold stratified selection of C over 0.01, 0.1, 1, and 10; secondary model fitted with prespecified C = 1.0 and assessed once in GSE52093. GSE52093 was not used for feature selection, tuning, or model choice.
**Receiver operating characteristic analysis**	pROC	pROC 1.18.5	Individual-gene and model ROC/AUC analyses in discovery and independent cohorts; threshold-selection settings not reported.
**Immune-cell deconvolution**	CIBERSORT R script; LM22 signature matrix	v1.03	LM22 (22 immune-cell types); between-group comparisons by two-sided Wilcoxon rank-sum test with Benjamini-Hochberg correction across 22 tests; gene-cell associations by Spearman correlation with Benjamini-Hochberg correction across 110 comparisons.

**Simulated gene knockout**	scTenifoldKnk	scTenifoldKnk 1.0.0	Simulated knockout analyses for *ANPEP*, *BCL3*, *BRD4*, *CCL20*, and *CD82*; additional settings not reported.
**Pseudotime trajectory analysis**	Monocle3	Monocle3 1.3.1	Monocyte trajectory and pseudotime analysis; *ANPEP*, *CCL20*, and *CD82* dynamics evaluated; root-cell and dimension settings not reported.
**Candidate compound screening**	DrugReflector	DrugReflector 1.0.0	Disease-associated signature screening; compounds prioritized by screening rank, biological relevance, docking feasibility, and structural availability; exact cutoffs not reported.
**Receptor and ligand preparation**	MGLTools	MGLTools 1.5.7	Receptor and ligand preparation for five selected compounds and five candidate targets; protonation and preparation settings not reported.
**Molecular docking**	AutoDock4; AutoGrid4	AutoDock4 4.2.6; AutoGrid4 4.2.6	AACOCF3, ruxolitinib, ML320, STK-249718, and SB-216763 docked against ANPEP, BCL3, BRD4, CCL20, and CD82; grid dimensions and search settings not reported.
**Spatial microenvironment analysis**	Custom R scripts; ggplot2	ggplot2 3.4.4; custom R scripts	GSE241870; module scores for smooth muscle cell contractile, endothelial, macrophage-like, and extracellular matrix-remodeling signals; descriptive use only because the targeted panel lacked the prioritized genes; additional settings not reported.
**ddPCR acquisition and quantification**	QX200 system; QuantaSoft	QuantaSoft version not reported	Each 22 µL reaction contained 11 µL EvaGreen Supermix, 100 nM primers, and 50-100 ng cDNA; cycling: 95°C for 5 min, 40 cycles of 95°C for 30 s and 60°C for 40 s, 4°C for 5 min, and 90°C for 5 min; Poisson-based absolute quantification; normalization to *GAPDH*; five paired biological experiments.
**Statistical analysis**	R base statistics and custom scripts	R 4.3.2	Benjamini-Hochberg correction where appropriate; two-sided $P < 0.05$; ddPCR paired-difference normality assessed by Shapiro-Wilk; paired t-test when approximately normal and paired Wilcoxon signed-rank test otherwise; data presented as mean ± SD.

Controlled exploratory fixed-gene analysis: ANPEP, CCL20, and CD82 fixed before resampling; L2 logistic regression; fold-specific standardization; outer leave-one-out cross-validation in GSE153434; inner three-fold stratified C selection over 0.01, 0.1, 1, and 10; secondary model fitted with prespecified C = 1.0 and assessed once in GSE52093; GSE52093 was not used for feature selection, tuning, or model choice. Historical exploratory machine-learning workflow retained for transparency only.

Supplementary Table S4. Integrated evidence for prioritized candidate genes

Evidence layer	*ANPEP*	*CCL20*	*CD82*	*BCL3*	*BRD4*
**Classification**	Leading candidate	Supporting candidate	Supporting candidate	Exploratory auxiliary gene	Exploratory auxiliary gene
**Bulk log2FC**	2.31	5.8	2.46	2.1	0.52
**Adjusted P value**	0.00703	0.000248	0.000617	0.00163	0.01499
**scPagwas PCC**	0.273	0.262	0.267	0.145	0.123
**hdWGCNA module**	Monocyte blue	Monocyte blue	Monocyte blue	Monocyte blue	Monocyte blue
**Module kME**	0.65	0.44	0.54	0.42	0.44
**Representative immune association**	Neutrophils: ρ = 0.74	Neutrophils: ρ = 0.71	Activated memory CD4 T cells: ρ = −0.70	Neutrophils: ρ = 0.49	Activated NK cells: ρ = −0.43
**Compound-screening candidate**	SB-216763	SB-216763	SB-216763	SB-216763	SB-216763
**Predicted docking energy (kcal/mol)**	-9.7	-7.38	-9.26	-8.08	-8.94

Supplementary Table S5. Full directional intersection gene lists

Monocyte blue-module intersection (module = blue; bulk DEG = up; scPagwas PCC = positive; n = 90)

Gene	*Module*	*Bulk DEG direction*	*scPagwas PCC*	*PCC sign*	*Included*
**AGO2**	blue	up	0.148	positive	True
**ANPEP**	blue	up	0.273	positive	True
**BCL3**	blue	up	0.145	positive	True
**BRD4**	blue	up	0.123	positive	True
**CAPZA1**	blue	up	0.132	positive	True

**Gene**	***Module***	***Bulk DEG direction***	***scPagwas PCC***	***PCC sign***	***Included***
**CCL20**	blue	up	0.262	positive	True
**CD82**	blue	up	0.267	positive	True
**CDC42**	blue	up	0.199	positive	True
**CDC42SE1**	blue	up	0.104	positive	True
**CXCL5**	blue	up	0.198	positive	True
**DOT1L**	blue	up	0.165	positive	True
**ECE1**	blue	up	0.120	positive	True
**EFHD2**	blue	up	0.188	positive	True
**ETS2**	blue	up	0.159	positive	True
**FAM129B**	blue	up	0.153	positive	True
**FGR**	blue	up	0.273	positive	True
**FMN1**	blue	up	0.121	positive	True
**FOSL2**	blue	up	0.137	positive	True
**FPGS**	blue	up	0.125	positive	True
**FPR1**	blue	up	0.241	positive	True
**FPR2**	blue	up	0.109	positive	True
**FTH1**	blue	up	0.226	positive	True
**FTH1P7**	blue	up	0.154	positive	True
**GAPDH**	blue	up	0.112	positive	True

**Gene**	***Module***	***Bulk DEG direction***	***scPagwas PCC***	***PCC sign***	***Included***
**GK**	blue	up	0.196	positive	True
**GNAI2**	blue	up	0.113	positive	True
**HIF1A**	blue	up	0.161	positive	True
**HIPK2**	blue	up	0.184	positive	True
**HK2**	blue	up	0.145	positive	True
**HMGA1**	blue	up	0.160	positive	True
**IL4R**	blue	up	0.148	positive	True
**IRAK2**	blue	up	0.189	positive	True
**IRAK3**	blue	up	0.183	positive	True
**ITGA5**	blue	up	0.139	positive	True
**KDM6B**	blue	up	0.102	positive	True
**LAPTM5**	blue	up	0.326	positive	True
**LHFPL2**	blue	up	0.114	positive	True
**LPCAT1**	blue	up	0.182	positive	True
**LUCAT1**	blue	up	0.147	positive	True
**MAP4K4**	blue	up	0.197	positive	True
**MAPK13**	blue	up	0.147	positive	True
**MARCO**	blue	up	0.216	positive	True
**MBOAT7**	blue	up	0.105	positive	True

Gene	*Module*	*Bulk DEG direction*	*scPagwas PCC*	*PCC sign*	*Included*
**MMP19**	blue	up	0.150	positive	True
**MXD1**	blue	up	0.200	positive	True
**MYD88**	blue	up	0.159	positive	True
**NAMPT**	blue	up	0.111	positive	True
**NEK6**	blue	up	0.110	positive	True
**NOD2**	blue	up	0.163	positive	True
**NRIP3**	blue	up	0.146	positive	True
**PGK1**	blue	up	0.140	positive	True
**PHC2**	blue	up	0.127	positive	True
**PIK3R5**	blue	up	0.273	positive	True
**PKM**	blue	up	0.109	positive	True
**PLAUR**	blue	up	0.208	positive	True
**PTPN1**	blue	up	0.181	positive	True
**PTPN12**	blue	up	0.107	positive	True
**RAB8B**	blue	up	0.115	positive	True
**RNF19B**	blue	up	0.139	positive	True
**S100A11**	blue	up	0.111	positive	True
**SBNO2**	blue	up	0.155	positive	True
**SEMA4B**	blue	up	0.116	positive	True

Gene	*Module*	*Bulk DEG direction*	*scPagwas PCC*	*PCC sign*	*Included*
**SEPT9**	blue	up	0.159	positive	True
**SERPINA1**	blue	up	0.308	positive	True
**SERPINB1**	blue	up	0.189	positive	True
**SERPINB8**	blue	up	0.132	positive	True
**SESN2**	blue	up	0.108	positive	True
**SIGLEC10**	blue	up	0.190	positive	True
**SLA**	blue	up	0.202	positive	True
**SLC11A1**	blue	up	0.255	positive	True
**SLC16A10**	blue	up	0.196	positive	True
**SLC16A3**	blue	up	0.224	positive	True
**SLC43A2**	blue	up	0.273	positive	True
**SLC6A6**	blue	up	0.217	positive	True
**SLC7A5**	blue	up	0.229	positive	True
**SMIM3**	blue	up	0.132	positive	True
**SOD2**	blue	up	0.117	positive	True
**SPP1**	blue	up	0.193	positive	True
**STAT3**	blue	up	0.202	positive	True
**STX4**	blue	up	0.113	positive	True
**TLR2**	blue	up	0.276	positive	True

Gene	*Module*	*Bulk DEG direction*	*scPagwas PCC*	*PCC sign*	*Included*
**TNFSF8**	blue	up	0.128	positive	True
**TRAF1**	blue	up	0.211	positive	True
**TREM1**	blue	up	0.259	positive	True
**TRMT6**	blue	up	0.101	positive	True
**UPP1**	blue	up	0.229	positive	True
**VDR**	blue	up	0.108	positive	True
**VHL**	blue	up	0.113	positive	True
**WTAP**	blue	up	0.116	positive	True
**ZMIZ1**	blue	up	0.102	positive	True

Smooth-muscle-cell turquoise-module intersection (module = turquoise; bulk DEG = down; scPagwas PCC = negative; n = 90)

Gene	*Module*	*Bulk DEG direction*	*scPagwas PCC*	*PCC sign*	*Included*
**ABCA8**	turquoise	down	-0.254	negative	True
**ABLIM1**	turquoise	down	-0.224	negative	True
**ACKR3**	turquoise	down	-0.200	negative	True
**ADAM33**	turquoise	down	-0.178	negative	True
**ADI1**	turquoise	down	-0.177	negative	True
**ALDH1A1**	turquoise	down	-0.139	negative	True
**ARHGAP21**	turquoise	down	-0.132	negative	True
**C3orf58**	turquoise	down	-0.120	negative	True

Gene	*Module*	*Bulk DEG direction*	*scPagwas PCC*	*PCC sign*	*Included*
**CALCRL**	turquoise	down	-0.171	negative	True
**CAV1**	turquoise	down	-0.202	negative	True
**CD34**	turquoise	down	-0.304	negative	True
**CD9**	turquoise	down	-0.105	negative	True
**CDKN1C**	turquoise	down	-0.164	negative	True
**CFI**	turquoise	down	-0.181	negative	True
**CFL2**	turquoise	down	-0.148	negative	True
**CHRDL1**	turquoise	down	-0.243	negative	True
**CLIC2**	turquoise	down	-0.159	negative	True
**COL12A1**	turquoise	down	-0.193	negative	True
**CRIM1**	turquoise	down	-0.127	negative	True
**CTSK**	turquoise	down	-0.208	negative	True
**CTSO**	turquoise	down	-0.154	negative	True
**CXCL14**	turquoise	down	-0.233	negative	True
**CYBRD1**	turquoise	down	-0.297	negative	True
**CYGB**	turquoise	down	-0.159	negative	True
**DAAM1**	turquoise	down	-0.114	negative	True
**DCLK1**	turquoise	down	-0.209	negative	True
**DLC1**	turquoise	down	-0.252	negative	True

**Gene**	***Module***	***Bulk DEG direction***	***scPagwas PCC***	***PCC sign***	***Included***
**DNAJB4**	turquoise	down	-0.227	negative	True
**DPT**	turquoise	down	-0.183	negative	True
**ECM2**	turquoise	down	-0.161	negative	True
**EFNB2**	turquoise	down	-0.179	negative	True
**EPB41L2**	turquoise	down	-0.180	negative	True
**FAM107A**	turquoise	down	-0.205	negative	True
**FEZ1**	turquoise	down	-0.129	negative	True
**FHL1**	turquoise	down	-0.181	negative	True
**FILIP1L**	turquoise	down	-0.195	negative	True
**FKBP9**	turquoise	down	-0.162	negative	True
**FOS**	turquoise	down	-0.251	negative	True
**FOXC1**	turquoise	down	-0.278	negative	True
**GEM**	turquoise	down	-0.276	negative	True
**GXYLT2**	turquoise	down	-0.138	negative	True
**HEG1**	turquoise	down	-0.207	negative	True
**ID4**	turquoise	down	-0.177	negative	True
**IGFBP6**	turquoise	down	-0.247	negative	True
**ISLR**	turquoise	down	-0.218	negative	True
**ITM2A**	turquoise	down	-0.250	negative	True

Gene	*Module*	*Bulk DEG direction*	*scPagwas PCC*	*PCC sign*	*Included*
**JUN**	turquoise	down	-0.209	negative	True
**KCTD9**	turquoise	down	-0.116	negative	True
**KLF2**	turquoise	down	-0.111	negative	True
**LIMA1**	turquoise	down	-0.260	negative	True
**LTBP1**	turquoise	down	-0.208	negative	True
**NDN**	turquoise	down	-0.189	negative	True
**NEGR1**	turquoise	down	-0.190	negative	True
**NTN4**	turquoise	down	-0.157	negative	True
**NTRK2**	turquoise	down	-0.189	negative	True
**OAT**	turquoise	down	-0.104	negative	True
**OGN**	turquoise	down	-0.267	negative	True
**OLFML3**	turquoise	down	-0.245	negative	True
**OMD**	turquoise	down	-0.238	negative	True
**OSBPL1A**	turquoise	down	-0.121	negative	True
**PAM**	turquoise	down	-0.186	negative	True
**PCYOX1**	turquoise	down	-0.142	negative	True
**PI16**	turquoise	down	-0.210	negative	True
**PID1**	turquoise	down	-0.121	negative	True
**PLS3**	turquoise	down	-0.213	negative	True

**Gene**	***Module***	***Bulk DEG direction***	***scPagwas PCC***	***PCC sign***	***Included***
**PLSCR4**	turquoise	down	-0.184	negative	True
**PPP2CB**	turquoise	down	-0.131	negative	True
**RAMP2**	turquoise	down	-0.230	negative	True
**RCN2**	turquoise	down	-0.130	negative	True
**SCARA5**	turquoise	down	-0.284	negative	True
**SFRP1**	turquoise	down	-0.157	negative	True
**SFRP4**	turquoise	down	-0.168	negative	True
**SGCB**	turquoise	down	-0.161	negative	True
**SH3D19**	turquoise	down	-0.175	negative	True
**SLIT2**	turquoise	down	-0.205	negative	True
**SLIT3**	turquoise	down	-0.165	negative	True
**SPTBN1**	turquoise	down	-0.243	negative	True
**SUN1**	turquoise	down	-0.132	negative	True
**TIMP3**	turquoise	down	-0.312	negative	True
**TMEM159**	turquoise	down	-0.113	negative	True
**TMEM176A**	turquoise	down	-0.158	negative	True
**TNFSF10**	turquoise	down	-0.145	negative	True
**TOB1**	turquoise	down	-0.125	negative	True
**TPM1**	turquoise	down	-0.127	negative	True

**Gene**	***Module***	***Bulk DEG direction***	***scPagwas PCC***	***PCC sign***	***Included***
**TSHZ2**	turquoise	down	-0.220	negative	True
**TWSG1**	turquoise	down	-0.156	negative	True
**UACA**	turquoise	down	-0.167	negative	True
**VGLL3**	turquoise	down	-0.194	negative	True
**VIT**	turquoise	down	-0.216	negative	True
**WEE1**	turquoise	down	-0.176	negative	True

The two 90-gene lists have no overlapping genes. PCC values are shown to three decimal places; included = True for every listed gene.

—, not evaluated in the independent cohort.
